# Nature-Inspired Upward Hanging Evaporator with Photothermal 3D Spacer Fabric for Zero-Liquid-Discharge Desalination

**DOI:** 10.1007/s40820-025-01868-0

**Published:** 2025-08-06

**Authors:** Ye Peng, Yang Shao, Longqing Zheng, Haoxuan Li, Meifang Zhu, Zhigang Chen

**Affiliations:** State Key Laboratory of Advanced Fiber Materials, College of Materials Science and Engineering, Donghua University, Shanghai, 201620 People’s Republic of China

**Keywords:** Desalination, Solar interfacial evaporation, Biomimetic design, Zero liquid discharge, Thermal management

## Abstract

While desalination is a key solution for global freshwater scarcity, its implementation faces environmental challenges due to concentrated brine byproducts mainly disposed of via coastal discharge systems. Solar interfacial evaporation offers sustainable management potential, yet inevitable salt nucleation at evaporation interfaces degrades photothermal conversion and operational stability via light scattering and pathway blockage. Inspired by the mangrove leaf, we propose a photothermal 3D polydopamine and polypyrrole polymerized spacer fabric (PPSF)-based upward hanging model evaporation configuration with a reverse water feeding mechanism. This design enables zero-liquid-discharge (ZLD) desalination through phase-separation crystallization. The interconnected porous architecture and the rough surface of the PPSF enable superior water transport, achieving excellent solar-absorbing efficiency of 97.8%. By adjusting the tilt angle (θ), the evaporator separates the evaporation and salt crystallization zones via controlled capillary-driven brine transport, minimizing heat dissipation from brine discharge. At an optimal tilt angle of 52°, the evaporator reaches an evaporation rate of 2.81 kg m^−2^ h^−1^ with minimal heat loss (0.366 W) under 1-sun illumination while treating a 7 wt% waste brine solution. Furthermore, it sustains an evaporation rate of 2.71 kg m^−2^ h^−1^ over 72 h while ensuring efficient salt recovery. These results highlight a scalable, energy-efficient approach for sustainable ZLD desalination.

**Supplementary Information:**

The online version contains supplementary material available at 10.1007/s40820-025-01868-0.

## Highlights

- Successful fabrication of photothermal 3D polypyrrole polymerized spacer fabric with excellent water transport capability and high solar absorption efficiency.
- The upward hanging model evaporator with reverse water feeding achieves an optimized solar evaporation rate of 2.81 kg m^−2^ h^−1^ with minimal heat (0.366 W) loss at a 52° tilt.
- A mangrove leaf-inspired upward hanging model evaporator design separates evaporation and crystallization zones for zero-liquid-discharge desalination.

**Supplementary Information:**

The online version contains supplementary material available at 10.1007/s40820-025-01868-0.

## Graphical abstract

**Figure:**
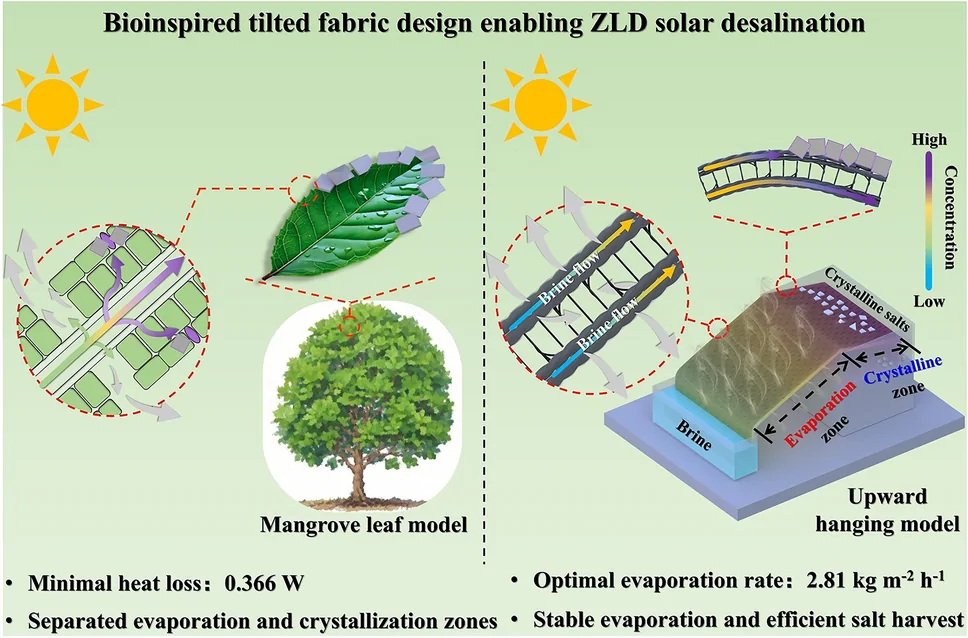

## Introduction

Desalination has demonstrated significant potential in addressing the pressing challenge of global freshwater scarcity, given that seawater is the most abundant water resource on Earth [1]. However, waste hypersaline brines (> 6 wt%) from desalination plants are often discharged into nearby lakes or seas, causing severe ecological damage [2, 3]. Despite being heralded as the epitome of sustainable desalination technology, zero-liquid-discharge (ZLD) systems confront significant technical and operational hurdles that impede their widespread implementation [4]. The conventional posttreatment approach is reverse osmosis (RO), or evaporating waste brine incurs escalating costs and specific energy consumption [5]. Therefore, developing an energy-efficient, cost-effective technology is imperative for sustainable ZLD desalination.

**Fig. 1:**
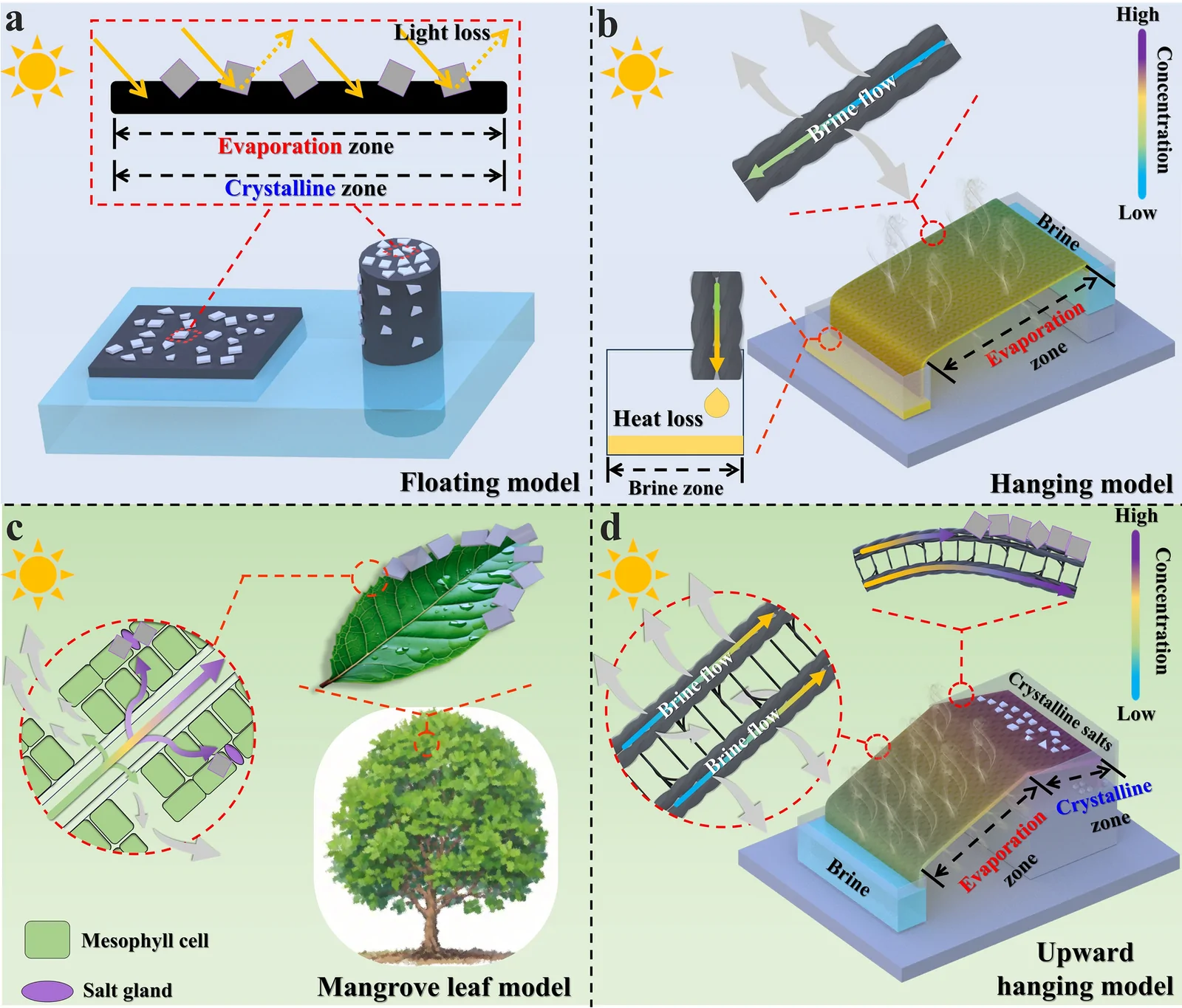
Conceptual biomimetic design of the 3D spacer fabric-based upward hanging model evaporator realizing ZLD desalination by a mangrove leaf. **a, b** Schematic illustration of floating and hanging model evaporators. **c** Transpiration and salt secretion effect of a mangrove leaf. **d** Schematic illustration of mangrove leaf-inspired upward hanging model evaporator

Solar-driven interfacial evaporation (SIE) has emerged as a promising sustainable technology, demonstrating unique potential to simultaneously address critical challenges in clean water production, renewable energy harvesting, and environmental remediation [6–8]. This technique harnesses the photothermal effect to evaporate water from seawater or hypersaline brines, showing great promise for ZLD desalination. Over the past few years, rapid advances in this field have spurred numerous studies aimed at enhancing the evaporation performance of floating models by improving light absorption [9–11], increasing photothermal conversion efficiency [12–15], expanding the effective evaporation area [16–19], and minimizing heat loss [20–23]. Nevertheless, rapid evaporation frequently leads to salt crystallization and accumulation in the evaporation zone (Fig. 1a), impeding light absorption and slowing vapor generation [24]. Recently, several studies have proposed rational designs, such as two-dimensional (2D) disks [10, 25], 2D flat [26–28], three-dimensional (3D) cups [29–31], 3D conical [32], and 3D spherical [33, 34] evaporators, which facilitate directional crystallization that isolates salt from the evaporation interface, thereby ensuring stable performance during treatment of hypersaline brines. However, current 2D evaporator designs suffer from low evaporation rates (0.5–1.42 kg m^−2^ h^−1^) due to limited single-surface areas, and although 3D evaporators show improved performance (1.26–3.26 kg m^−2^ h^−1^), their complex and costly fabrication processes hinder large-scale deployment. To address these issues, our group recently proposed a double-surface hanging model using low-cost, easy-to-prepare, and scalable photothermal fabrics suspended between two water tanks (Fig. 1b) [35–42]. During operation, brine infiltrates the fabric and flows downward via capillary and siphoning effects. Most of the brine evaporates from both surfaces of the fabric, boosting the evaporation rate (1.8–2.3 kg m^−2^ h^−1^) compared to the 2D evaporators, while the remaining concentrated brine drips off in to the collection tank, preventing salt accumulation in the evaporation zone. The hanging evaporation device, however, generates approximately 0.2 kg of high-concentration brine (near saturation point) per kilogram of water evaporated, creating potential environmental contamination hazards through saline discharge [39]. Furthermore, the inherent thermal conductivity of water (≈ 0.6 W m^−1^ K^−1^) induces substantial parasitic heat dissipation through brine transport in evaporation systems, resulting in efficiency degradation in solar-driven evaporation processes [42]. Hence, one of the next objectives in SIE toward ZLD lies in engineering scalable evaporators that simultaneously suppress salt crystallization at evaporation area, maintain a high evaporation rate, and facilitate salt resource recovery.

Mangroves provide an ideal natural model for ZLD through stomatal transpiration and salt gland secretion (Fig. 1c). In mangrove leaves, stomatal transpiration generates negative pressure to drive the upward movement of water and salt ions, while salt glands excrete ions that crystallize along leaf edges to maintain ion balance [43]. Inspired by this natural mechanism, we developed an upward hanging model for ZLD desalination. In our design, a photothermal fabric is suspended between two water tanks at different elevations, forming a folded structure (Fig. 1d). By precisely adjusting the upward tilt angle (θ), brine travels upward along the photothermal fabric through capillary, allowing most of the water to rapidly evaporate from the upper evaporation zone without brine discharge, while salt ions migrate and crystallize exclusively in a designated lower segment, effectively separating the evaporation and crystallization zones. Furthermore, to enhance light absorption and expand the evaporation area, we present a photothermal 3D spacer fabric prepared via in situ polymerization of polydopamine (PDA) and polypyrrole (PPy) on spacer fabric (PPSF). Benefiting from its light-trapping architecture, our PPSF attains broad-spectrum (280–2500 nm) absorption with a solar-absorbing efficiency of 97.8%. When θ is 52°, the upward hanging model minimizes heat loss to 0.366 W and yields an optimized evaporation rate of 2.81 kg m^−2^ h^−1^ under 1-sun illumination while treating a 7 wt% waste brine solution. Moreover, the model maintains an evaporation rate of 2.71 kg m^−2^ h^−1^ and facilitates salt recovery over three consecutive days. These findings represent a promising route for next-generation sustainable desalination systems that integrate advanced photothermal materials with nature-inspired design.

## Experimental Section

### Fabrication of SF

Polyethylene terephthalate (PET) fibers were knitted into a 3D spacer fabric (designated as SF) using a semiautomatic warp knitting machine. During weaving, twisted interlaced PET yarns with an average diameter of 900 μm (single fiber diameter: ~ 15 μm) were knitted on the front and back needle bars to form the top and bottom fabric layers, while PET fibers with an average diameter of 50 μm were alternately knitted on the front and back needle bars to create a middle spacer layer connecting the top and bottom substrates. The as-prepared fabric (thickness: 0.3 cm) was immersed in a ternary solvent system (acetone/ethanol/deionized water = 1:1:1 v/v) and sonicated for 30 min, then rinsed three times with deionized water to remove surface impurities. Finally, the pretreated fabric was dried in an oven at 45 °C for subsequent use. The large-scale SF was customized by Jieyingtu New Materials Technology Co., Ltd, Shanghai, China.

### Preparation of PPSF

First, the pretreated SF was modified via in situ polymerization of polydopamine (PDA). Specifically, 0.10 g tris(hydroxymethyl)aminomethane was dissolved in 100 mL deionized water and stirred thoroughly, and the solution was adjusted to pH =8.5 using HCl. Then, 0.15 g dopamine hydrochloride was added and stirred until the solution changed from colorless to light brown. The SF was immersed in this solution and continuously stirred at room temperature for 12 h. Afterward, the PDA-modified SF (designated as PSF) was dried at 60 °C for 1 h and rinsed three times with deionized water. Next, the PSF was further modified to incorporate polypyrrole (PPy). 0.55 g polyvinyl alcohol (PVA) and 600 μL pyrrole were dissolved in 90 mL deionized water and stirred for 30 min to form a PVA/pyrrole solution. The PSF fabric was then immersed in a FeCl₃·6H₂O solution (0.8 mol L^−1^) for 1 h to adsorb sufficient ferric ions. Subsequently, the ferric-decorated fabric was immersed in the above PVA/pyrrole solution and stirred for another 30 min. The entire mixture was then allowed to react at 4 °C for 12 h. Finally, the resulting PPy-modified PSF (designated as PPSF) was dried at 60 °C for 1 h, removed, and rinsed three times with deionized water. For comparison, traditional 2D fabric without spacer structure was also treated with PDA and PPy to obtain photothermal fabrics (designated as PPF) (thickness: 0.1 cm).

### Construction of Upward Hanging Model Evaporator

An upward hanging model was constructed to realize ZLD desalination by spatially separating the evaporation and crystallization zones (Fig. 1d). Two polymethyl methacrylate (PMMA) tanks at different elevations served as a saline inlet (lower) and a salt collection reservoir (upper). A PPSF was suspended in a folded configuration, rising from the lower reservoir to an apex for evaporation before angling downward into the upper reservoir. By adjusting the height difference between the tanks, the upward tilt angle (θ) of the fabric can be precisely tuned to regulate water flow. The fabric was secured with magnetic clips under ambient conditions, and external disturbances were minimized. During operation, both the evaporation and crystallization zones are illuminated simultaneously, and the evaporation rate is calculated based on their combined projected area (Fig. S1). Details on materials, characterization, and additional experimental procedures are provided in Supplementary Section S1.

## Results and Discussion

### Preparation and Characterization of PPSF

**Fig. 2:**
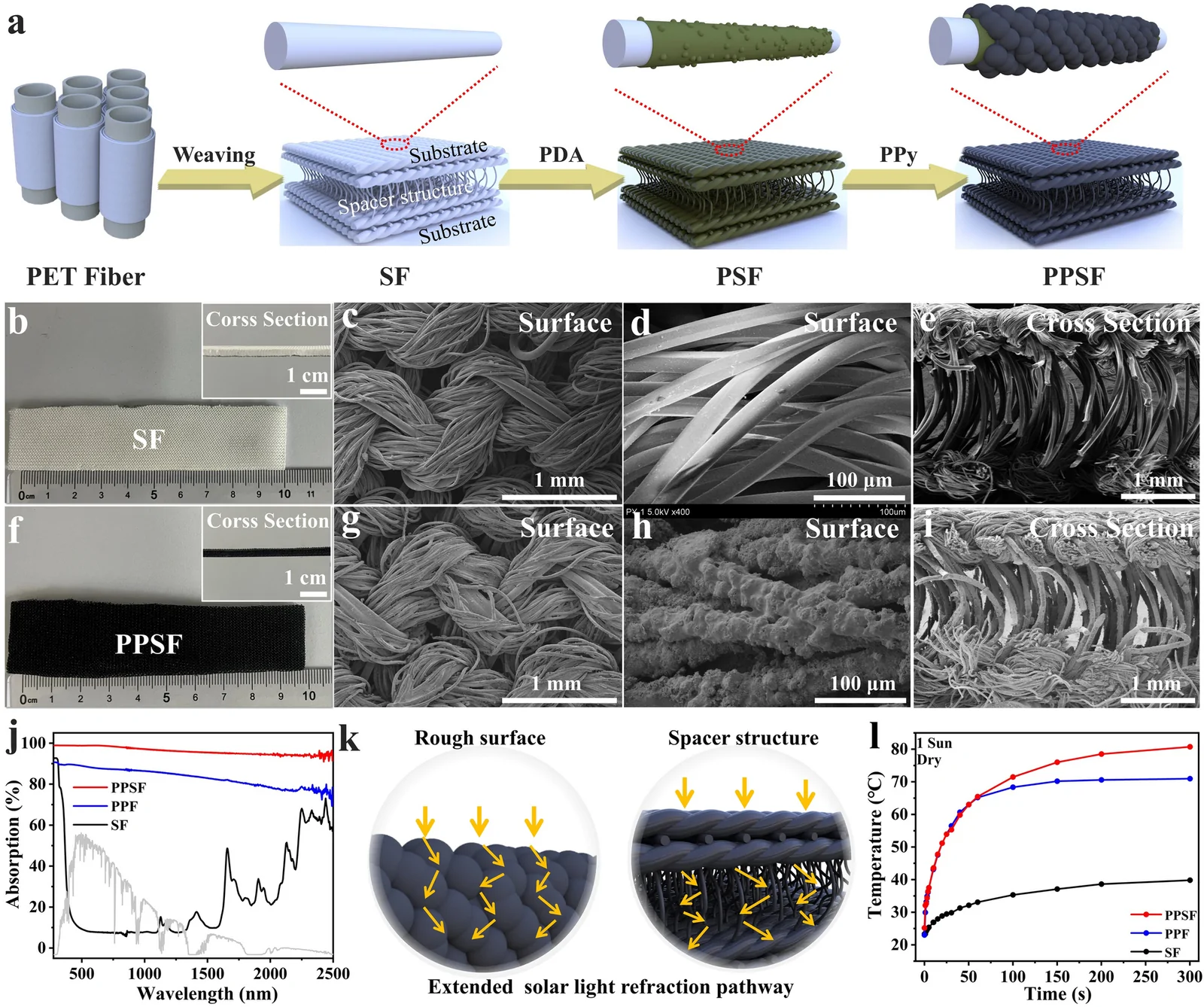
Preparation and characterization of PPSF.** a** Schematic diagram for preparing the PPSF. **b** Digital photographs and **c–e** SEM images of the SF. **f** Digital photos and **g–i** SEM images of the PPSF. **j** UV–Vis–NIR spectra of fabrics in the wavelength ranging from 280 to 2500 nm. **k** Schematic illustration of high-efficiency light absorption based on rough surface and spacer structure of PPSF. **l** Temperature curves of the dry fabrics under solar illumination (1 kW m^−2^)

As shown in Fig. 2a, the 3D knitted PET spacer fabric (SF) was developed using a semiautomatic knitting machine. The overall structure of the white SF consists of two separate knitted substrates that are kept apart by spacer yarns (Fig. 2b). In the surface view (Fig. 2c, d), the smooth/clean knitted substrates are composed of fiber bundles with a diameter of ~ 900 µm. From the side-sectional view (Fig. 2e), the dense substrates are formed by fiber bundles, while the loose intermediate spacer is composed of smooth-surface fibers with a diameter of ~ 50 μm and a length of ~ 1.5 mm. To further improve the wetting ability of the SF fabric and endow it with a photothermal conversion effect, we prepared the PPSF through a two-step self-polymerization process involving dopamine and pyrrole. After the first-step growth of PDA on the pristine SF fabric, the fabric turned brown (Fig. S2a). The surface of the pristine fibers became rough as they were partly covered by nanoparticles, indicating that the PDA was successfully decorated on the fibers (Fig. S2b–d). After the second-step modification with PPy, the color of the fabric turned dark (Fig. 2f). These fibers were completely covered by a coarse outer layer, which results in a distinct increase of diameter from ~ 15 to ~ 20 µm (Fig. 2g, h). Notably, the PPSF retains the spacer structure woven by the knitting machine (Fig. 2i). In addition, the elemental mapping images of PPSF indicated the presence of C, O, N, and Cl elements (Fig. S3). The Cl likely originates from FeCl_3_, which is used in the reaction to form PDA, and N may derive from both PDA and PPy, suggesting successful loading of PDA and PPy onto the fabric. The strong binding between the fabric and the PDA/PPy layer is ensured through covalent and non-covalent interactions, providing stability and durability (Fig. S4) [44–46]. The overall structure of PPSF is analogous to a leaf, with the upper and lower surfaces functioning like xylem to facilitate solution transport, and the middle connecting region acting like stomata to enhance the release of steam. This bionic design provides an excellent structural foundation for evaporation and mass transport.

### Interaction of PPSF with Light

The optical absorptions of SF and PPSF were analyzed using ultraviolet–visible–near infrared (UV–Vis-NIR) spectroscopy over the range of 280–2500 nm. For comparison, traditional 2D fabric without spacer structure was also modified by PDA/PPy (PPF) and tested under same conditions (Fig. S5). From these spectra (Fig. S6a, b), the absorption spectra were calculated according to the previous report [42]. Obviously, the initial white-color SF demonstrated feeble light absorption capability and a low degree of solar-absorbing efficiency at 12.5% (Fig. 2j). The deep-black PPF had an improved solar-absorbing efficiency of ~ 89.0%, due to the loading of photothermal materials. Significantly, within the wavelength range of 280–2500 nm, PPSF exhibited low reflectance (< 5%) and transmittance (< 10%), with its solar-absorbing efficiency amounting to 97.8%. This enhanced and broad-spectrum optical absorption is attributed to the rough surface of the fibers and the complex structure of the fabric, which prolong the light refraction path within the material (Fig. 2k).

The photothermal conversion of SF, PPF, and PPSF was evaluated using an infrared camera under 1-sun illumination (Fig. 2l). The dry SF revealed a slow temperature climb, starting at 22 °C and ending at 39.0 °C after 300 s. Conversely, the dry PPF and PPSF both experienced quick temperature rises within 50 s, and the equilibrium temperature of PPSF at 300 s reached 81.2 °C, which is 11.3 °C higher than that of PPF (70.5 °C). These findings demonstrate that the PPSF architecture possesses superior photothermal conversion efficiency, thereby significantly advancing solar-driven evaporation performance.

### The Interaction of PPSF with Water

**Fig. 3:**
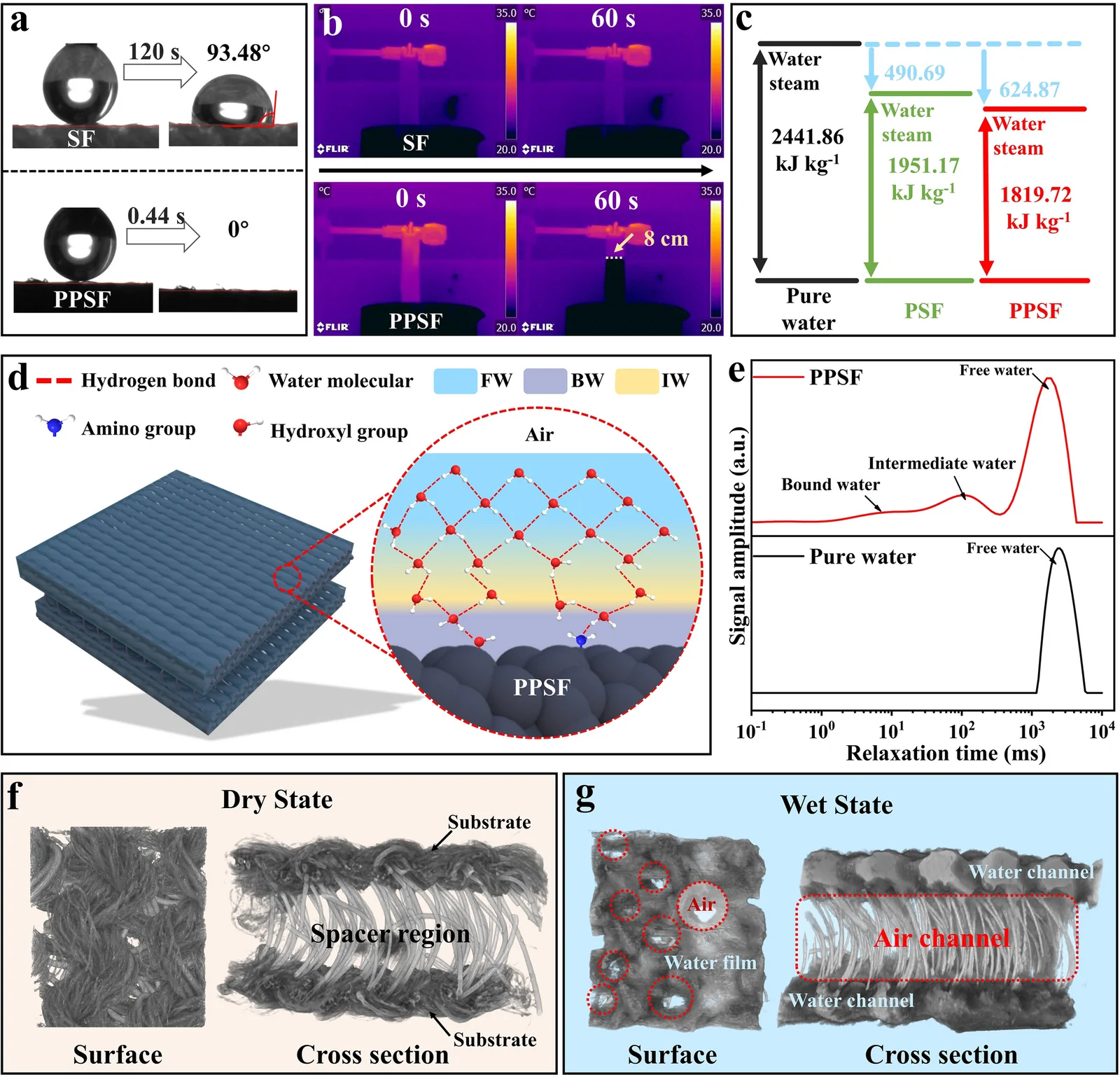
Interaction of PPSF with water. **a** Shapes of a water droplet on the surface of SF and PPSF. **b** Water-soaking behavior of SF and PPSF. **c** Equivalent evaporation enthalpy of bulk water, water in PSF and PPSF, respectively. **d** Schematic diagram of the water states inside PPSF. **e** LF-NMR spectrum of bulk water and water inside PPSF. **f, g** Water and air distribution in PPSF during the transition from dry to wet state

The surface functional groups and hydrophilic characteristics of photothermal materials critically govern hydration dynamics and evaporation thermodynamics [47]. To examine these interfacial properties, Fourier-transform infrared spectroscopy (FTIR) was systematically employed to characterize chemical modifications between SF and PPSF (Fig. S7). In the spectrum of PPSF, two new absorption peaks appeared at the 1260 and 1505 cm^−1^, which were attributed to the N–H bending vibration and C-N stretching vibration of amines (-NH_2_) of PDA and PPy [48, 49]. In addition, the new absorption peak at 3391 cm^−1^ corresponded to the -OH stretching vibration of the oxhydryl (-OH) in PDA [50, 51]. The rich function groups (-OH and -NH_2_) may endow the PPSF with strong hydrophilicity. Surface wettability evolution was quantitatively characterized through dynamic contact angle measurements (Figs. 3a and S8). The pristine SF exhibited persistent hydrophobicity with a contact angle of 93.5° showing no temporal decay (0% reduction over 120 s), indicative of non-wetting characteristics. On the contrary, PSF and PPSF demonstrated instantaneous liquid imbibition with contact angle decrease to 0° within 0.76 and 0.44 s, respectively, confirming superhydrophilic transformation. To further investigate the water-soaking behavior, the water penetration front along the length of the samples was tracked by infrared imaging (Fig. 3b). The wicking height of PPSF reached 8 cm after 60 s of passive capillary pumping. This result indicates that PPSF has the capability of efficient water supply.

Previous studies have demonstrated that the presence of abundant hydrophilic functional groups can significantly reduce evaporation enthalpy. Suppose that the evaporation is driven by the same energy input (*U*_in_) according to Yu’s report [47, 52], the evaporation enthalpies can be determined as below.

1 $${U}_{\mathrm{in}}=\Delta {H}_{\mathrm{w}}{\dot{m}}_{\mathrm{w}}=\Delta {H}_{\mathrm{ev}}{\dot{m}}_{\mathrm{ev}}$$

where $\Delta {H}_{\mathrm{w}}$ (2441.86 kJ kg^−1^ at 25 °C [53]) and $\Delta {H}_{\mathrm{ev}}$ are the evaporation enthalpies of bulk water and water-soaked fabric samples, respectively. ${\dot{m}}_{\mathrm{w}}$ and ${\dot{m}}_{\mathrm{ev}}$ refer to the measured mass change rates of bulk water and fabric samples. The bulk water and water-soaked fabrics with the identical surface areas were synchronously tested in a desiccator with hygroscopic silica gel and recorded the mass change rate under dark conditions (25 °C, 45% RH) (Fig. S9a). The dark mass change rates of PSF and PPSF were 27.41 g m^−2^ h^−1^ and 29.39 g m^−2^ h^−1^, respectively, both surpassing that of bulk water at 21.08 g m^−2^ h^−1^ (Fig. S9b). The ${\Delta H}_{\mathrm{ev}}$ of the water in PSF and PPSF was calculated using Eq. (1), yielding 1951.17 and 1819.72 kJ kg^−1^, respectively. Both values are lower than the enthalpy of pure water (2441.86 kJ kg^−1^) (Fig. 3c). Notably, the PPSF exhibits the lowest evaporation enthalpy (1819.72 kJ kg^−1^), on account of its abundant hydrophilic moieties (-OH and -NH_2_) to establish enhanced hydrogen bonding and electrostatic interactions with water molecules. These strong interfacial interactions, which in turn lead to the generation of a bound water (BW) layer near the PPSF surface, consequently lower the energy barrier for water evaporation (Fig. 3d). In the outer layer of BW, water molecules are coordinated with less than four other water molecules, creating the intermediate water (IW) layer. The IW layer is associated with reduced energy requirements for the processes of breaking hydrogen bonds and escaping from the liquid surface, ultimately causing a reduction in the enthalpy of water evaporation [54]. The hydrated layer can be formed between BW and IW layers, with free water (FW) molecules populating the outermost periphery. To quantify the proportion of IW, low-field nuclear magnetic resonance (LF-NMR) was performed on wetted PPSF and bulk water. LF-NMR tracks the transverse relaxation of ^1^H nuclei, where differences in molecular mobility produce distinct T₂ domains corresponding to bound water (< 10 ms), intermediate water (10–100 ms), and free water (> 1000 ms), enabling quantification of each fraction [55, 56]. While pure water exhibits a singular relaxation peak at 2492 ms (characteristic of FW mobility), the wetted PPSF displays three peaks: a primary peak at 1693.3 ms corresponding to FW (peak area proportion: 67.5%), secondary peak at 91.6 ms representing IW (peak area proportion: 33.2%), and a residual BW signal at 3.9 ms (peak area proportion: 0.3%) (Fig. 3e). These results confirm that the hydrophilic groups in PPSF remarkably boost the IW proportion, thereby reducing the energy required for water to transition from a liquid state to a gaseous state.

Additionally, the proper distribution of the water and air can increase the evaporation area by forming new liquid/air interfaces in the photothermal materials, thereby enhancing the evaporation rate [57, 58]. To investigate the distribution of the water and air, the nano-computed tomography (nano-CT) was employed to observe the structure of the PPSF before and after wetting. Compared with the spacer fabric in the dry state, water was attached to the top and bottom substrates in the form of a thin water film on the wetted PPSF, while a considerable amount of air was retained to form air channels in the intermediate spacer region (Fig. 3f, g). This unique regionalized distribution of water and air enables both the upper and lower surface of substrates to be in contact with air, further expanding the evaporation area of PPSF.

### Solar-Powered Evaporation of Upward Hanging Model Evaporator

In conventional floating evaporation systems, the bottom surface of the evaporator remains submerged in bulk water, restricting phase-change processes exclusively to the upper air–liquid interface (Fig. S10a). In contrast, a hanging model is constructed by suspending a fabric in air and immersing its two edges into separate tanks (one filled with brine and the other empty). This configuration establishes dual evaporation interfaces along both air-exposed surfaces while effectively localizing thermal energy generated by solar absorption within these optimized evaporation zones, thereby significantly enhancing thermal efficiency through interfacial heating confinement (Fig. S10b). To further enhance the evaporation performance of the hanging model, we developed a PPSF with hierarchical structure. The PPSF, exhibiting strong photoabsorption and photothermal properties along with low evaporation enthalpy, enables rapid light-induced liquid–vapor phase transitions. More importantly, due to the regionalized distribution of water and air on the fabric, both the upper and lower substrates of PPSF are in contact with air, further forming a four-sided evaporation (Figs. 3g and S10c). To confirm above concept, the hanging PPSF was tested under an irradiated solar simulator (1.0 kW m^−2^). The surface temperatures of the wet PPSF were monitored in real-time using an infrared camera, and the mass change was measured with an electronic balance. For comparison, the hanging evaporator (PPF), floating evaporator (PPF), and brine (7 wt%) were also tested under the same conditions. As shown in Fig. S10d, the brine solution showed a minimal temperature rise from 21.0 to 22.7 °C within 0–600 s. In contrast, as the floating evaporator (PPF) and hanging evaporator (PPF) were exposed to sunlight, the surface temperatures of the evaporators rose from 21.0 to 36.6 and 37.6 °C, respectively. Notably, the hanging evaporator (PPSF) reached the highest surface temperature, with temperatures rising rapidly from 21.0 to 39.9 °C within 0–600 s. This is attributed to the enhanced photoabsorption of the PPSF, facilitated by its light-trapping effect (Fig. 2j). Regarding the evaporation rate, as shown in Fig. S10e, f, the brine solution (7 wt%) and floating evaporator (PPF) displayed low evaporation rates of 0.30 and 1.13 kg m^−2^ h^−1^, respectively. The hanging evaporator (PPF) showed an increased evaporation rate of 1.55 kg m^−2^ h^−1^. Remarkably, the hanging evaporator (PPSF) attained the highest evaporation rate of 1.93 kg m^−2^ h^−1^, which was 6.43, 1.71, and 1.25 times higher than the brine solution (7 wt%), floating evaporator (PPF), and hanging evaporator (PPF), respectively.

**Fig. 4:**
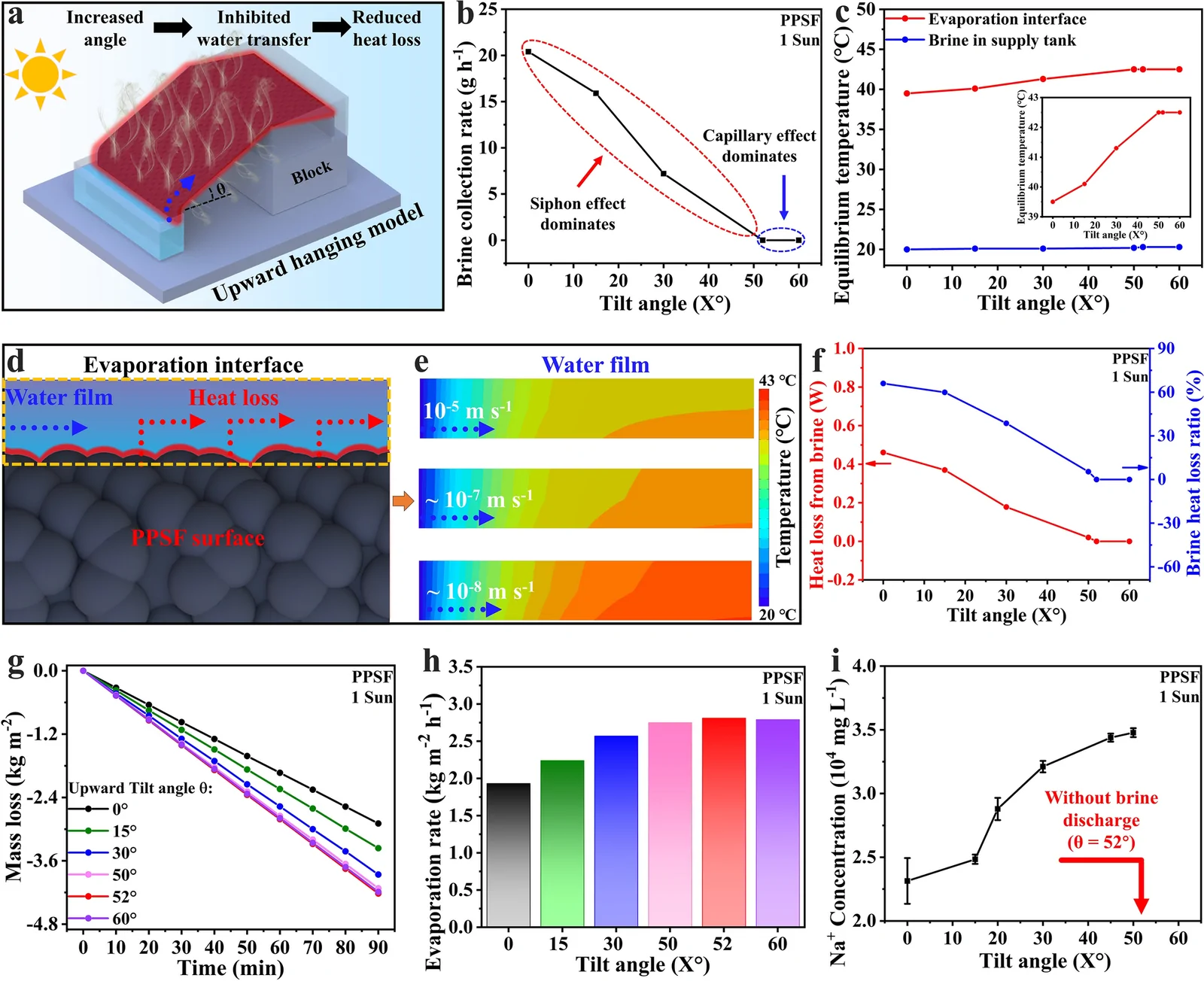
Impact of tilt angle of PPSF on the upward hanging model evaporator performance. **a** Schematic of the upward hanging model, showing reduced water transfer and heat loss with increased tilt angle. **b, c** Brine collection rate, equilibrium temperature of the evaporation interface and brine in supply tank at various tilt angles of PPSF. **d–f** Illustration and COMSOL simulation results of heat loss due to flowing water. **g, h** Mass loss and evaporation rate at various tilt angles.** i** Na⁺ concentration in the collected brine at different tilt angles, demonstrating higher concentration at higher angles and zero liquid discharge at 52°

A neglected phenomenon in hanging evaporators is that rapid water flow can carry heat away from the fabric surface to the collection tank, thereby reducing the photothermal evaporation rate. To enhance thermal energy utilization, properly regulating water transport is essential. This can be achieved by adjusting the fabric tilt angle (θ) via rationally controlling the height difference between the two tanks (Fig. 4a). When the fabric is tilted upward, water transfer decreases due to gravity and the reduced siphon effect (Fig. S11a). To explore the effect of fabric tilt angle on the heat loss from brine flow under 1 sun of irradiation, the PPSF was tilted to various angles, and the mass change of collected tank was recorded using an electronic balance. Meanwhile, the temperatures of the wet evaporation interface and the simulated brine in the supply tank were measured using an infrared camera. As shown in Fig. 4b, when the fabric was horizontal (θ = 0°), the mass change of collected tank was 3.4 g within 10 min, corresponding to a fast brine collection rate of 20.4 g h⁻^1^. As the fabric was tilted upward to 15°, 30°, and 50°, the siphon effect gradually diminished, resulting in decreased brine collection rates of 15.9, 7.2, and 0.8 g h^−1^, respectively. Notably, when the fabric was further tilted to 52°, no water was transferred to the empty collection tank, as the capillary effect dominated water supply rather than the siphon effect (Fig. S11b). Figure 4c shows that the temperature of the simulated brine in supply tank remained at approximately 20 °C during the solar evaporation process. This result should be attributed to the low thermal conductivity of the PPSF, which effectively prevent heat transfer to the bulk water (Fig. S12). It should be noted that when the tilt angles (θ) were changed from 0° to 60°, the equilibrium temperatures of the evaporation interface increased from ~ 39.5 to ~ 42.5 °C, respectively. These results reveal that increasing the tilt angle leads to an increase in the evaporation interface temperature, which is attributed to the retarded water transfer within the fabric. This effect may potentially enhance water evaporation. To further analyze the heat transfer behavior, evaporation interfacial models (comprising a flowing water film and PPSF) with different water flow rates were constructed using COMSOL (Fig. 4d). As the same energy was input into the PPSF, the flowing water films possessed different temperature gradients. Specifically, when the water flow rate was ~ 10^–5^ m s^−1^, the flowing water film had a temperature gradient increasing from 20 °C at the water inlet to a highest temperature of 39 °C (Fig. 4e). When the water flow rates were further decreased from ~ 10^–7^ to ~ 10^–9^ m s^−1^, the highest temperature of the flowing water films increased from 40 to 42 °C. This may be attributed to the increased contact time between water molecules and the photothermal interface when the water flow rate decreases, leading to more efficient heat exchange and an elevated temperature at the water film [59].

Based on the above measured data, the heat loss from brine flow (*Q*_brine flow_) of the evaporators at various angles can be estimated as follows:

2 $$Q_{{\text{brine flow}}} = v \cdot C \cdot (T_{{e{\text{vaporation surface}}}} - T_{{{\mathrm{brine}}}} )$$

where *v* is the brine collection rate (g h^−1^), *C* is the specific heat capacity of water (4.2 J g^−1^ K^−1^),* T*_evaporation surface_ is the equilibrium temperature of evaporation interface, and *T*_brine_ is the equilibrium temperatures of brine in supply tank. As shown in Fig. 4f and Table 1, when the fabric was horizontal (θ = 0°), the heat loss was highest due to the fastest water transfer. The *Q*_brine flow_ was 0.461 W, accounting for 66.0% of the total heat loss (*Q*_total loss_ = 0.698 W). As the tilt angle increased to 15°, 30°, and 50°, *Q*_brine flow_ decreased to 0.370, 0.178, and 0.05 W, corresponding to approximately 59.8%, 38.7%, and 5.3% of the total heat loss, respectively. These results are mainly attributed to a reduction in the brine collection rate as the tilt angle increases. Notably, when the fabric was tilted at 52°, no heat loss from water flow was achieved, as no brine was transferred to the empty tank, and the evaporation rate reached its maximum value of 2.81 kg m^−2^ h^−1^, which was 1.46 times higher than that at 0° (1.93 kg m^−2^ h^−1^) (Fig. 4g, h). However, at tilt angle of 60°, the evaporation rate slightly decreased to 2.79 kg m^−2^ h^−1^. This is due to enhanced thermal convective heat, resulting in a higher total heat loss (*Q*_total loss_ = 0.416 W) compared to the tilt angle of 52° (*Q*_total loss_ = 0.366 W). These results confirm that adjusting the fabric tilt angle effectively reduces heat loss and boosts the performance of the hanging evaporator. With the tilt angle optimized, wind-field tests (Fig. S13) revealed that directing cross-winds of 1 and 2 m s^−1^ onto the side surface of PPSF elevated the evaporation rate from 2.81 kg m^−2^ h^−1^ (0 m s^−1^) to 3.40 kg m^−2^ h^−1^ and 4.32 kg m^−2^ h^−1^, respectively, confirming that external airflow can further enhance evaporation.

**Table 1:** The heat loss of the evaporator at different tilt angles

θ (X°)	v (g h^−1^)	T_brine_ (K)	T_evaporation surface_ (K)	Q_brine flow_ (W)	Q_total loss_ (W)	Q_brine flow_ ratio (%)
0	20.4	~ 293.15	~ 312.65	0.461	~ 0.698	66.0
15	15.9	~ 293.15	~ 313.25	0.370	~ 0.619	59.8
30	7.2	~ 293.15	~ 314.45	0.178	~ 0.460	38.7
50	0.8	~ 293.15	~ 315.65	0.02	~ 0.377	5.3
52	0	~ 293.15	~ 315.65	0	~ 0.366	0
60	0	~ 293.15	~ 315.65	0	~ 0.416	0

Along with the water that evaporated, the residual brine present on the fabric became concentrated. As the tilt angle rose, the concentration of the collected brine became higher due to slower water supplementation and faster water evaporation. As shown in Fig. 4i, when the fabric was horizontal (θ = 0°), the Na⁺ concentration of the collected brine was comparatively low (2.31 × 10^4^ mg L^−1^), nearly matching the original simulated brine (2.11 × 10^4^ mg L^−1^). As the tilt angle increased to 50°, the measured Na⁺ concentration reached 3.47 × 10^4^ mg L^−1^. Notably, when the tilt angle was further increased to 52°, no brine was transferred to the collection tank, achieving zero liquid discharge and thus avoiding environmental threats posed by brine discharge.

### Salt Crystallization Behavior of the Upward Hanging Model Evaporator

**Fig. 5:**
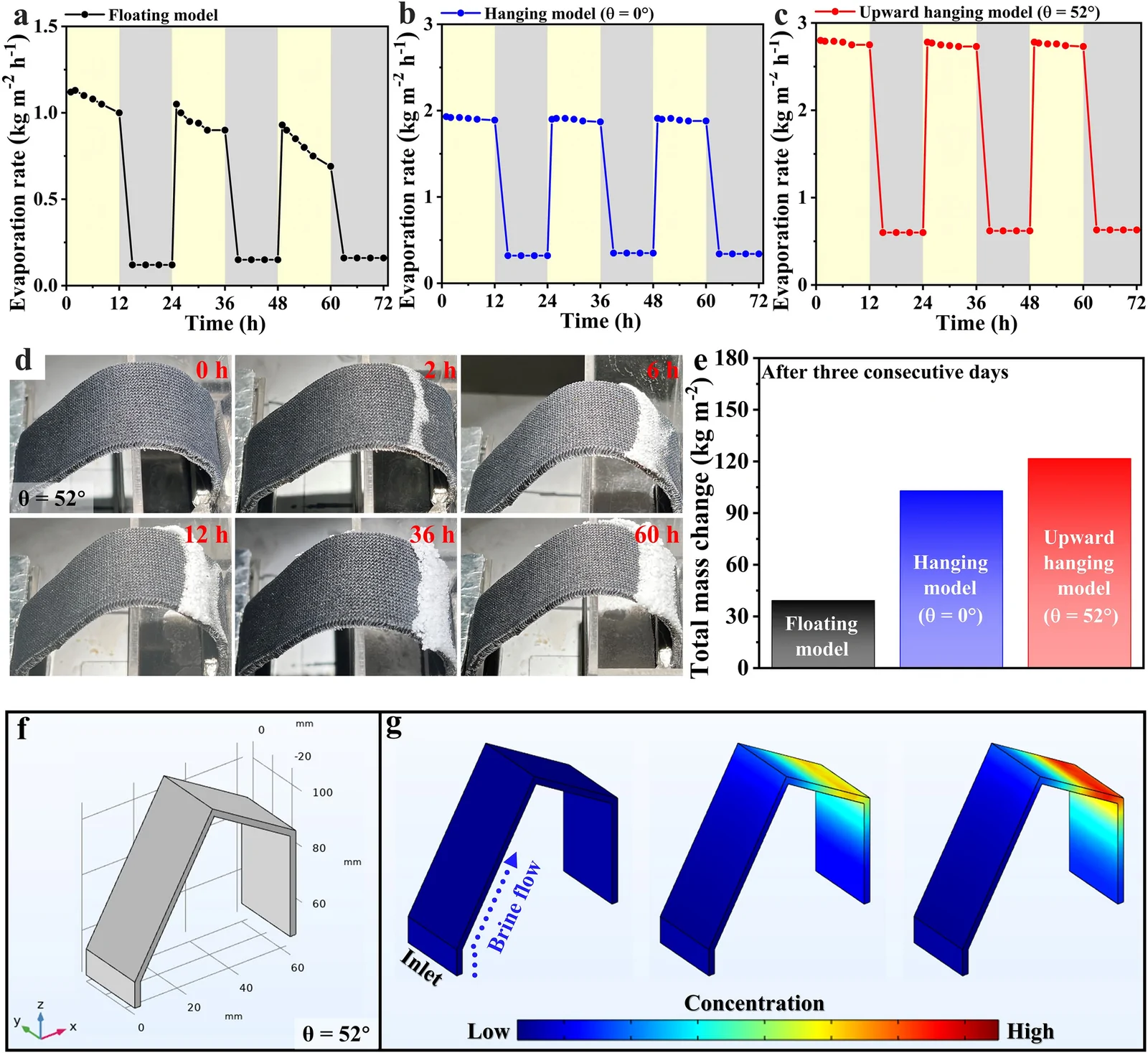
Durability and salt crystallization behavior of upward hanging model evaporator. **a–c** Long-term evaporation of floating, hanging (θ = 0°) and upward hanging (θ = 52°) models during three consecutive days using brine solution (7 wt%). **d** Photographs of the upward hanging model (θ = 52°) during long-term evaporation, illustrating salt accumulation at the lower segment without affecting the evaporation zone. **e** Total evaporation mass of evaporation models after three consecutive days. **f–g** COMSOL simulated distribution of salt ion concentration over the upward hanging model (θ = 52°) during evaporation

Severe salt crystallization often occurs during the evaporation process of solar desalination, limiting the continuous use of solar evaporators. To investigate the durability, the upward hanging model (θ = 52°) was used to evaporate brine solution (7 wt%) for three consecutive days, with 12 h of simulate solar illumination (1 kW m^−2^) and 12 h of dark environment each day. For comparison, the floating model and the hanging model (θ = 0°) were tested under the same conditions. The solar evaporation rate of the floating model was measured to be 1.12 kg m^−2^ h^−1^ at the beginning of the first simulated day and gradually decreased to 0.68 kg m^−2^ h^−1^ by the end of the last simulated day (Fig. 5a). A large amount of solid salt crystallized on the fabric surface was compared to the fabric before the test (Fig. S14a). In contrast, the hanging model (θ = 0°) showed stable solar and dark evaporation rates of approximately 1.90 and 0.35 kg m^−2^ h^−1^, respectively, without salt crystallization (Figs. 5b and S14b). For the upward hanging model (θ = 52°), the solar and dark evaporation rates further increased and stabilized at approximately 2.71 and 0.62 kg m^−2^ h^−1^, respectively (Fig. 5c). Meanwhile, salt gradually accumulated and was limited to the lower segment of the PPSF in collected tank, ensuring the effective area of the evaporation zone and maintaining stable photothermal conversion and high-efficiency four-sided evaporation over three consecutive days (Fig. 5d). After testing, the upward hanging model (θ = 52°) demonstrated the best evaporation performance, with a total mass loss of 121.68 kg m^−2^, which was 3.10 times higher than that of the floating evaporator (39.24 kg m^−2^) (Fig. 5e). Additionally, in the upward hanging model (θ = 52°), part of the accumulated salt falls into the collection tank due to gravity, while residual salt on the fabric can be easily removed using tweezers (Fig. S15). During three consecutive days of operation, 7.284 g of salt crystals was collected from the upward hanging model evaporator (Fig. S16). After 14 consecutive days of evaporation, the breaking strength of post-service PPSF in the dry state is 5.2 MPa, indicating its mechanical robustness for handling, transport, and prolonged use (Fig. S17). Following a manual washing process, the post-service PPSF also retained solar-absorbing efficiency of 93%, only 4.9% lower than that of pristine PPSF (97.8%), and the corresponding evaporator still delivered an evaporation rate of 2.70 kg m^−2^ h^−1^, only 3.9% below initial value (2.81 kg m^−2^ h^−1^) (Fig. S18). These experimental results demonstrate that the upward hanging model can simultaneously achieve long-term high efficiency, stable steam production, and continuous salt collection.

Interestingly, the salt crystallization phenomenon on the hanging evaporator can be regulated by adjusting the fabric tilt angle (θ). When the tilt angle was 0°, the evaporator exhibited salt-free evaporation. This is attributed to the continuous infiltration of salt water into the fabric and its eventual arrival in the collection tank. This process transfers salt ions from the fabric surface to the collection tank, preventing the salt concentration on the fabric surface from reaching saturation (Fig. S19). When the tilt angle increased to 52°, the evaporator achieved a clear separation between the evaporation and crystallization zones, ensuring stable water evaporation and efficient salt recovery. This phenomenon can be explained as follows: As evaporation progresses, a concentration gradient of salt develops within the folded configuration of the fabric. The dissolved salt migrates upward and then accumulates along the downward-tilted surface, eventually reaching supersaturation and crystallizing. Thus, geometry of the design and orientation of the system direct water to the upward-tilted evaporation zone while confining salt accumulation to the downward-tilted crystallization zone. COMSOL simulations (Fig. 5f, g) corroborate this mechanism by predicting high ion concentrations in the downward-tilted surface and relatively low ion concentrations in upward-tilted surface, confirming that evaporation and crystallization occur in spatially distinct regions.

### Practical Performance of the Upward Hanging Model Evaporator

**Fig. 6:**
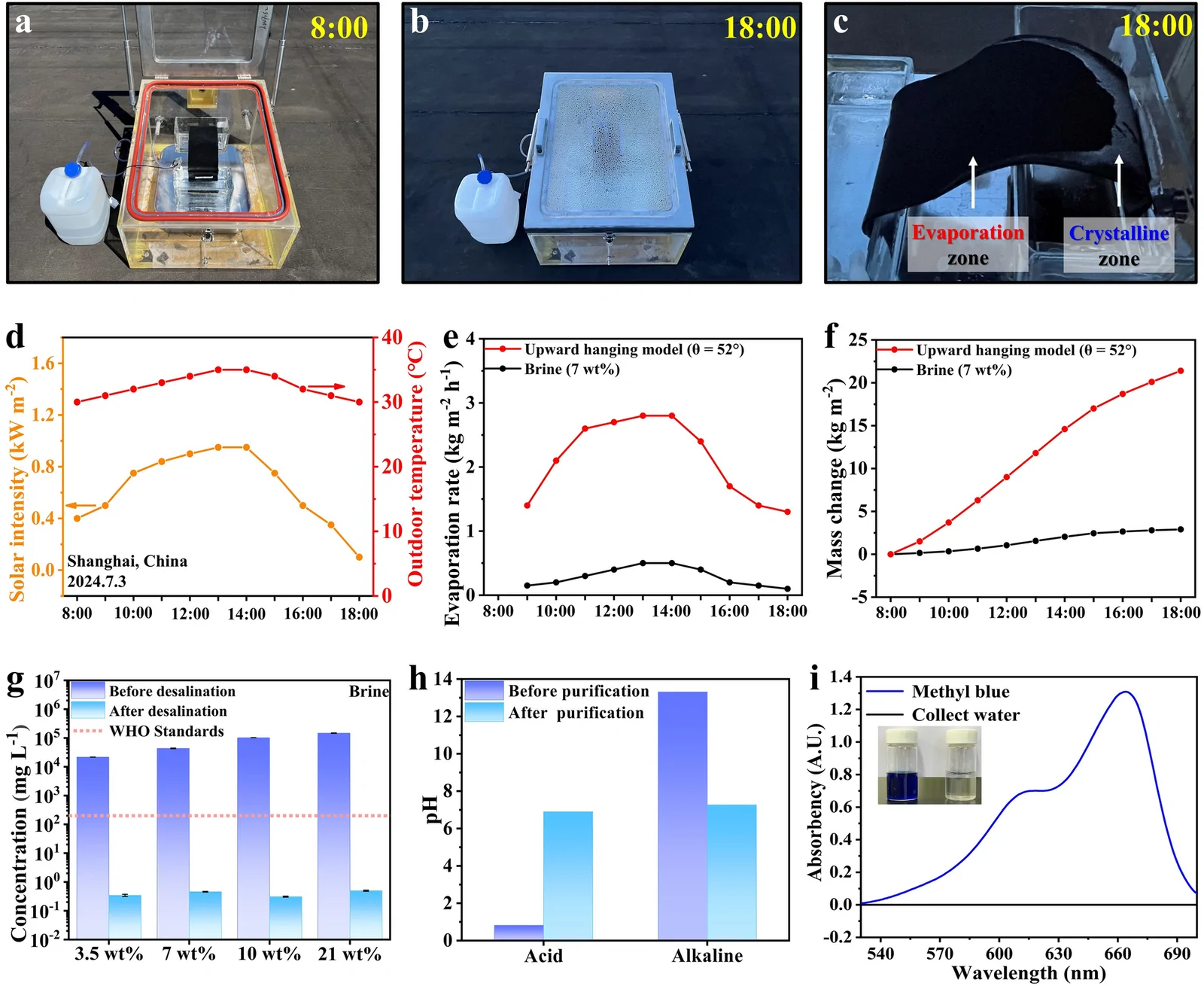
Practical performance of the upward hanging model evaporator. **a, b** Photographs of the device for freshwater collection before and after operation. **c** Photographs of the surface of PPSF after the one-day operation. **d** Change of solar intensity and outdoor temperature on July 3, 2024. **e, f** Evaporation rates and the accumulated mass change of the upward hanging model (θ = 52°) and brine at various day times.** g** Ion concentrations in brine before and after treatment. **h** Change in pH value of acid and alkaline solutions before and after purification. **i** Photographs and photoabsorption spectra of methyl blue solutions before and after purification

The evaporation performance of the evaporator under natural sunlight is significant for ZLD treatment in practical applications. Furthermore, the preparation process of PPSF involves the semi-automated knitting technology and straightforward polymerization strategies, which is suitable for the large-scale production of flexible photothermal fabric with good portability (Fig. S20). Herein, large PPSF (project area: 200 cm^2^) was equipped into upward hanging model evaporator (θ = 52°), containing brine solution (7 wt%) and put in a condenser (Fig. 6a). The produced vapor experienced condensation at the top region of the transparent tilted device and was directed to flow into the bottom of the device (Fig. 6b). The incident solar intensity, outdoor temperature, and weight losses of evaporator from 8:00 to 18:00 were recorded. For comparative purposes, an outdoor experiment using real brine was also conducted. As the solar intensity gradually increased from 0.41 kW m^−2^ at 8:00 to 0.95 kW m^−2^ at 13:00, the outdoor temperature rose from 30.6 to 35.4 °C. When the solar intensity further went down to 0.1 kW m^−2^ at 18:00, the outdoor temperature also decreases to 30.0 °C (Fig. 6d). Consequently, the evaporation rate for the brine gradually increased from 0.15 kg m^−2^ h^−1^ at 9:00 to 0.5 kg m^−2^ h^−1^ at 13:00, and then declined to 0.1 kg m^−2^ h^−1^ at 18:00 (Fig. 6e). Similarly, the evaporation rate for the evaporator also changed from 1.4 kg m^−2^ h^−1^ at 9:00 to 2.8 kg m^−2^ h^−1^ at 13:00, and then reduced to 1.3 kg m^−2^ h^−1^ at 18:00. After the 10 h evaporation, the accumulated mass change of the evaporator reached 21.4 kg m^−2^, which is 7.4 times higher than that (2.9 kg m^−2^) of the brine (Fig. 6f). Furthermore, a portion of the generated steam was condensed and collected, with the water production mass of 28.1 g (Fig. S21). Additionally, the evaporation and crystallization zones on the PPSF surface showed spatial separation (Fig. 6c), indicating the significant potential of the upward hanging model evaporator for large-scale water and salt production under natural condition.

Our suspended system achieves dual functionality in sustainable brine management: while executing high-efficiency brine-free discharge treatment, it simultaneously enables freshwater production through integrated steam condensation modules. As shown in Fig. 6g, the Na^+^ concentration in the collected freshwater (< 10 mg L^−1^) was significantly lower than those in the original brine solutions at different concentration. These concentrations are well below the taste threshold set by the World Health Organization (WHO). Additionally, the upward hanging model was used to treat acid and alkaline liquids. The purified water samples demonstrate pH values close to neutral (Fig. 6h). Furthermore, liquid sample containing methyl blue was also purified. As shown in Fig. 6i, the purified water obtained through solar evaporation was colorless and transparent, with no characteristic absorption peaks of methyl blue (667 nm) present. These results demonstrate that the solar evaporation process using the upward hanging model effectively removes salt ions and dye pollutants, and the collected evaporated water can be used to prepare pure water.

## Conclusion

This study presents a nature-inspired three-dimensional spacer photothermal fabric (PPSF) evaporator engineered for ZLD desalination applications. The biomimetic evaporator demonstrates exceptional photothermal conversion capabilities, achieving excellent solar-absorbing efficiency of 97.8% through its hierarchical architecture that enables prolonged light-trapping via multiple internal reflections. Innovatively adopting mangrove-inspired transpiration principles, we designed an upward hanging evaporation system where the vertically folded PPSF membrane bridges two tanks at differential elevations. This configuration establishes distinct functional zones: continuous vapor generation occurs at the elevated segment while concentrated brine undergoes directional salt crystallization in the lower chamber, effectively eliminating liquid waste discharge. Under optimized conditions (52° tilt angle, 1-sun irradiation), the system attains an evaporation rate of 2.81 kg m^−2^ h^−1^ with remarkable thermal confinement (0.366 W heat loss) when treating a 7 wt% waste brine solution. Notably, the evaporator maintains operational stability over extended periods, demonstrating a sustained evaporation rate of 2.71 kg m^−2^ h^−1^ and efficient salt recovery (1.62 kg m^−2^ day^−1^) without performance degradation. This dual-functional design simultaneously addresses freshwater production and salt harvesting challenges, representing a paradigm-shifting approach to sustainable desalination technology development. The demonstrated synergy between bioinspired structural engineering and interfacial solar thermal management paves the way for practical implementation of energy-efficient ZLD systems.

## Supplementary Information

Below is the link to the electronic supplementary material.

Supplementary file1 (DOCX 7347 KB)
