# Supplementary material for: Nature-Inspired Upward Hanging Evaporator with Photothermal 3D Spacer Fabric for Zero-Liquid-Discharge Desalination
Source: Nanomicro Lett. 2025 Aug 6;18:22. doi: 10.1007/s40820-025-01868-0 (PMC12325164; doi:10.1007/s40820-025-01868-0)
Supplement: Supplementary file 1 — Supplementary file1 (DOCX 7347 KB) [file 40820_2025_1868_MOESM1_ESM.docx]

Supporting Information for

**Nature-Inspired Upward Hanging Evaporator with Photothermal 3D Spacer Fabric for Zero-Liquid-Discharge Desalination**

Ye Peng^1,#^, Yang Shao^1,#^, Longqing Zheng^1^, Haoxuan Li^1,^ *, Meifang Zhu^1^, Zhigang Chen^1,^ *

^1^State Key Laboratory of Advanced Fiber Materials, College of Materials Science and Engineering, Donghua University, Shanghai 201620, P. R. China

^#^Ye Peng and Yang Shao contributed equally to this work.

* Corresponding authors: E-mail: [hxli@dhu.edu.cn](mailto:hxli@dhu.edu.cn) (Haoxuan Li); [zgchen@dhu.edu.cn](mailto:zgchen@dhu.edu.cn) (Zhigang Chen)

**S1 Experiment Details**

**S1.1 Chemicals and materials**

Dopamine hydrochloride (DA, C_8_H_11_NO_2_∙HCl, Mw: 189.34, 98%) was purchased from Aladdin. Pyrrole (C_4_H_5_N, CP, ≥98.0%), Tris(hydroxymethyl)aminomethane (C_4_H_11_NO_3_, 98%), Hydrochloric acid (HCl, 36.0 ~ 38.0 wt%) and FeCl_3_∙6H_2_O (AR) were purchased from Sinopharm Chemical Reagent Co., Ltd. Poly (vinyl alcohol) (PVA, model 1788) was obtained from Aladdin.

**S1.2 Characterizations**

The morphologies and functional groups of samples were characterized by field emission scanning electron microscopy equipped with energy dispersive spectroscopy (FE-SEM, Hitachi S-4800). The reflectance R(λ) and transmittance T(λ) were tested by UV-Vis-NIR reflectance spectroscopy (Lambda 950, PerkinElmer) in the range of 280-2500 nm. The absorbance A(λ) was calculated by Kirchhoff’s law:

$A\left( \lambda\right)=1-R(\lambda)-T(\lambda)$ (S1)

The solar-absorbing efficiency (α) were calculated by integrating the absorption spectra normalized to AM1.5 G standard spectrum.

$a=\frac{\int_{280 nm}^{2500 nm} \left( 1-R\left( \lambda\right)-T(\lambda) \right)G\left( \lambda\right)d\lambda}{\int_{280 nm}^{2500 nm} G\left( \lambda\right)d\lambda}$ (S2)

where $G\left( \lambda\right)$ is the standard AM 1.5 G spectral irradiance.

The water contact angles of the materials were analyzed by contact angle meter (CA, SL200KS). The spin-spin relaxation times (T_2_) of samples were determined using low-filed nuclear magnetic resonance spectrometer (LF-NMR, Niumai VTMR20-010V-I). The structure of the sample before and after wetting was observed by nano-computed tomography (Nano-CT, NanoVoxel-300). The concentrations of metal ion in the brine and the collected freshwater were tested by inductively coupled plasma atomic emission spectrometry (ICP-AES, Prodigy).

**S1.3 Estimation of water evaporation enthalpy at 25 ℃**

We use the Eq. (S3) to calculate the $\Delta H_{w}$ [S1].

$\Delta H_{w}=\alpha+\beta T+\gamma T^{1.5}+\delta T^{2.5}+\varepsilon T^{3}$ (S3)

Where $T$ is the equilibrium temperature (℃) of the evaporation interface, $\alpha$ = 2500.304, $\beta$ = -2.2521025, $\gamma$ = -0.021465847, $\delta$ = 3.1750136×10^-4^, $\varepsilon$ = -2.8607959×10^-5^ are constants. Thus, the $\Delta H_{w}$ of pure water at 25 ℃ was calculated to be 2441.86 kJ kg^−1^.

**S1.4 Indoor solar evaporation experiments**

Indoor solar evaporation experiments were conducted by fixing fabric samples (2.5 cm × 15 cm) between two PMMA tanks at varying tilt angles (θ = 0, 15, 30, 50, 52 and 60°). The spacing between the two tanks was maintained at 3 cm, with the fabric end positioned 1 cm above the bottom of the collection tank. A platform balance beneath the tanks continuously recorded weight changes over time. The fabric was exposed to vertical irradiation from a solar simulator (PL-X300D, 300 W) with a uniform square-shaped light spot (8 cm × 8 cm) at an intensity of 1.0 kW m^-2^, measured using an optical power meter (Newport, Model 1918-C, CA, USA). The surface temperature of the fabric was monitored using an infrared camera (A300, FLIR Systems Inc.). The evaporation rate was determined by dividing the weight loss rate (kg h^-1^) by the projected illumination area (15 cm^2^). For comparison, a floating model was constructed by placing a PPF fabric (5 cm × 3 cm) on a PS foam support, which was floated on the water surface for evaporation tests. The test conditions were: room temperature of ~25 ℃, and humidity of ~45%.

**S1.5 Estimation of heat loss**

**Table S1** The heat loss of the evaporator at different tilt angle

| θ  (X°) | *Q_brine flow_*  (W) | *Q_conv_*  (W) | *Q_rad_*  (W) | *Q_cond_*  (W) | *Q_total loss_*  (W) |
| --- | --- | --- | --- | --- | --- |
| 0 | 0.461 | 0.237 | 1.03×10^-7^ | ~ 0 | ~ 0.698 |
| 15 | 0.370 | 0.249 | 1.07×10^-7^ | ~ 0 | ~ 0.619 |
| 30 | 0.178 | 0.282 | 1.25×10^-7^ | ~ 0 | ~ 0.460 |
| 50 | 0.02 | 0.357 | 1.62×10^-7^ | ~ 0 | ~ 0.377 |
| 52 | 0 | 0.366 | 1.64×10^-7^ | ~ 0 | ~ 0.366 |
| 60 | 0 | 0.416 | 1.92×10^-7^ | ~ 0 | ~ 0.416 |

The total heat loss (*Q_total loss_*) of evaporator can be estimated as follows [S2, S3]:

$\text{Q}_{\text{total loss}}\text{=}\text{Q}_{\text{conv}}\text{+}\text{ }\text{Q}_{\text{rad}}\text{+}{\text{ }\text{Q}}_{\text{cond}}\text{+}\text{ }\text{Q}_{\text{brine flow}}$ (S4)

The convection heat loss (*Q_conv_*) takes place from evaporator to ambient air is expressed as:

$\text{Q}_{conv}\text{=}h\text{ }\text{∙}\text{A}\text{ }\text{∙}\text{ }\text{(}T_{evaporation surface}\text{- }T_{ambient air}\text{)}$ (S5)

Where *h* is the heat transfer coefficient (⁓ 5 W/m^2^), *A* is the photothermal evaporation surface area of PPSF, *T_evaporation surfac_*_e_ is the equilibrium temperatures of fabric's evaporation surface.

The radiative energy transfer (*Q_rad_*) from evaporator to ambient environment can be evaluated as:

$\text{Q}_{rad}\text{=}\varepsilon\text{∙ }\sigma\text{ ∙ A ∙ }\text{(}\text{T}_{\text{evaporation surface}}^{\text{4}}\text{- }\text{T}_{\text{ambient air}}^{\text{4}}\text{)}$ (S6)

Where ε represents the emissivity of the evaporation surface (≈0.9), σ is the Stefan-Boltzmann constant (5.67×10^-8^ W m^-2^ K^-4^).

The conduction heat loss (*Q_cond_*) happens from PPSF to the NaCl solution and can evaluated as:

$\text{Q}_{cond}\text{=}\text{ }\text{C∙}\text{M }\text{∙}\text{ }\text{∆T }$ (S7)

where *C* is the specific heat capacity of water (4.2 J g^−1^ K^−1^), *M* is the weight of the brine solution (100 g) and Δ*T* is the temperature change of the bulk water.

The heat loss from brine (*Q_water flow_*) can be evaluated as:

$\text{Q}_{\text{brine flow}}\text{=v}\text{ }\text{∙}\text{ }\text{C}\text{ }\text{∙}\text{ }\text{(}T_{evaporation surface}\text{- }T_{brine}\text{)}\text{ }$ (S8)

Where *v* is the brine collection rate (g h^-1^), *C* is the specific heat capacity of water (4.2 J g^−1^ K^−1^), *T_brine_* is the temperatures of brine in the supply tank.

**S1.6 Outdoor solar evaporation**

The outdoor evaporation experiments were conducted in transparent condenser on a sunny day, July 3, 2024. The large-scale PPSF was hung between two PMMA water tanks and tilted the fabrics at 52° (project area: 200 cm^2^). A platform balance (model AR-223) was positioned to accurately measure the mass changes. To monitor the environmental conditions, a light intensity meter was utilized to record the solar irradiance, and a thermometer was employed to track the outdoor temperature.

**S2 Supplementary Figures**

**
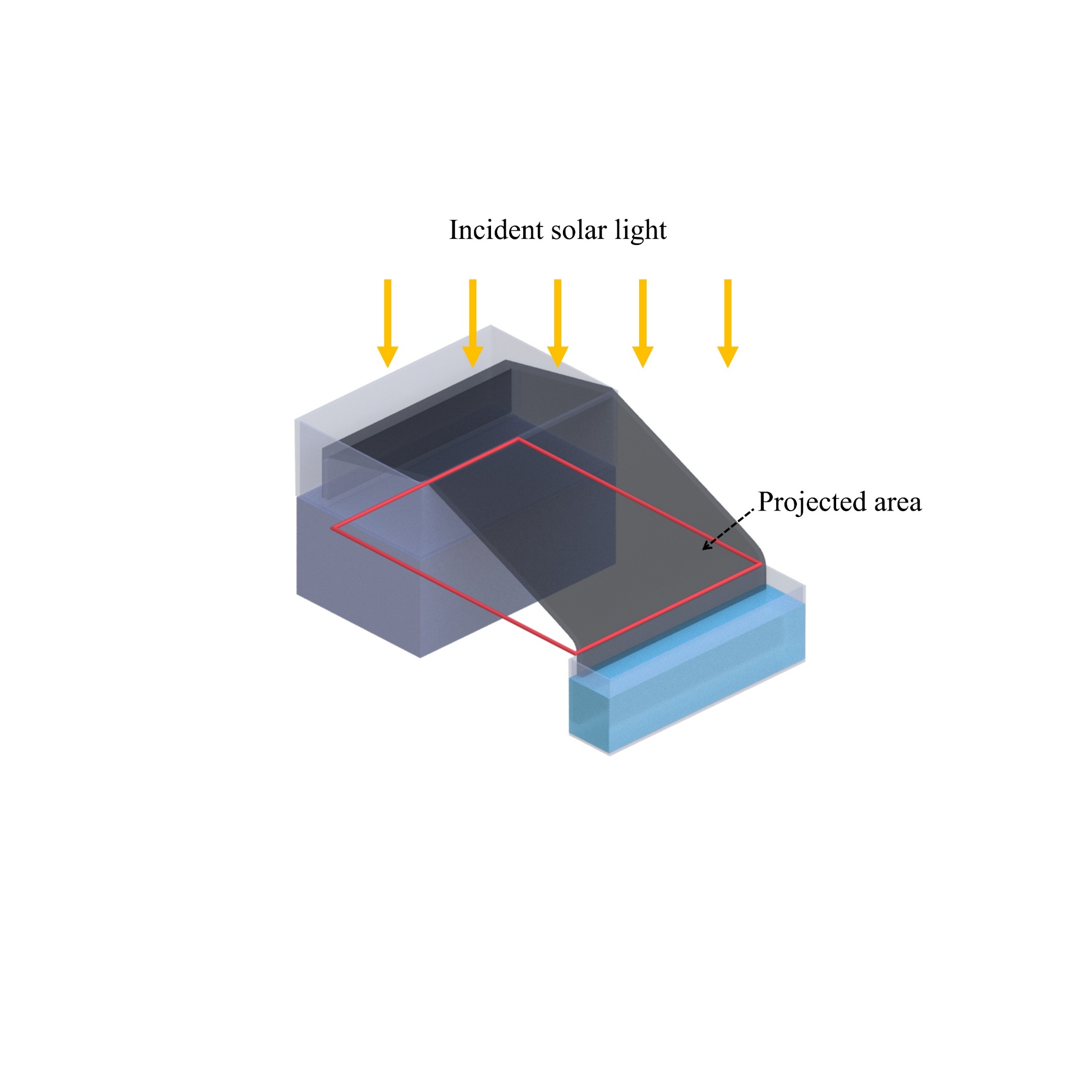
**

**Fig. S1** Schematic illustrations of the projected area of upward hanging evaporator


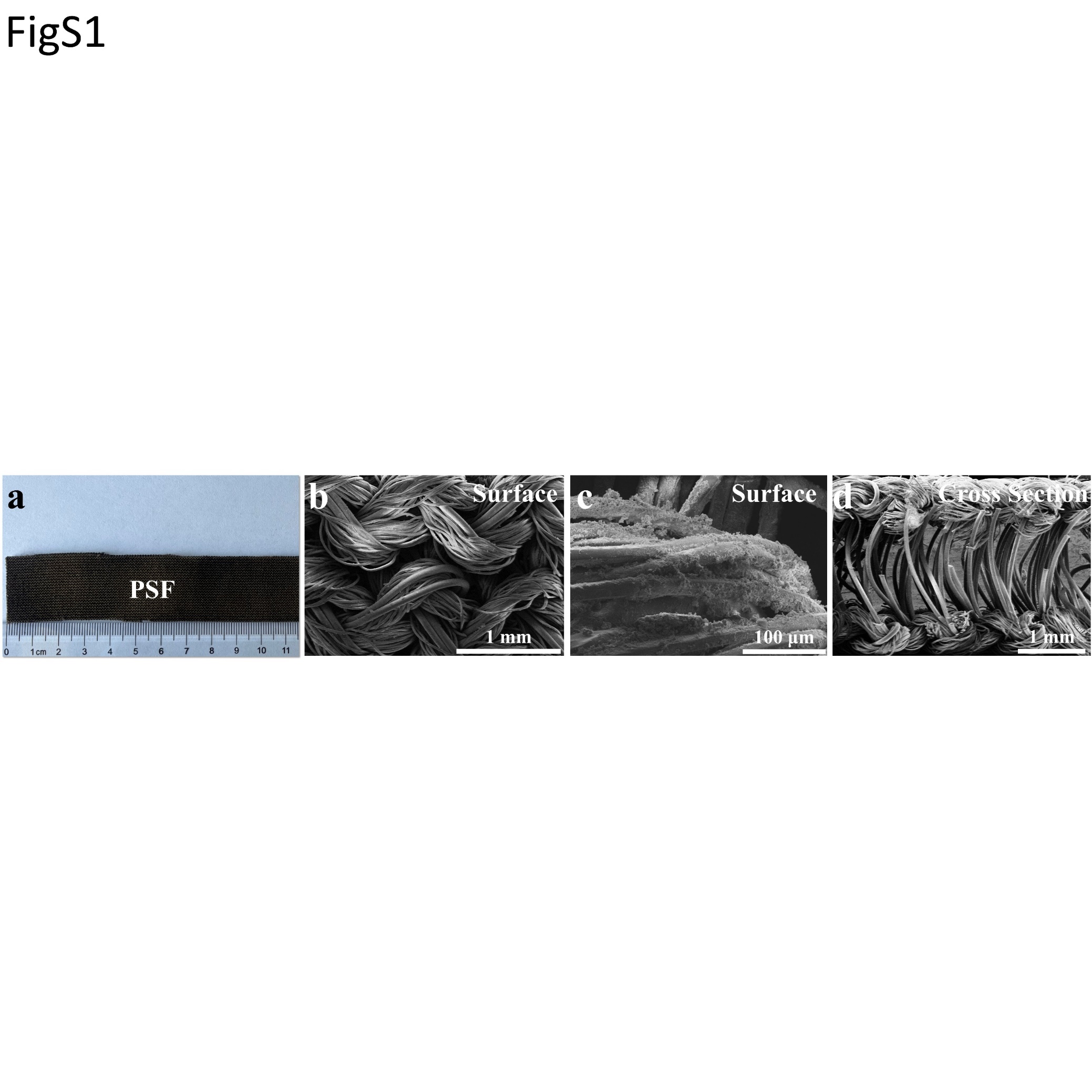


**Fig. S2 a** Digital photos and **b-d** SEM images of the PSF


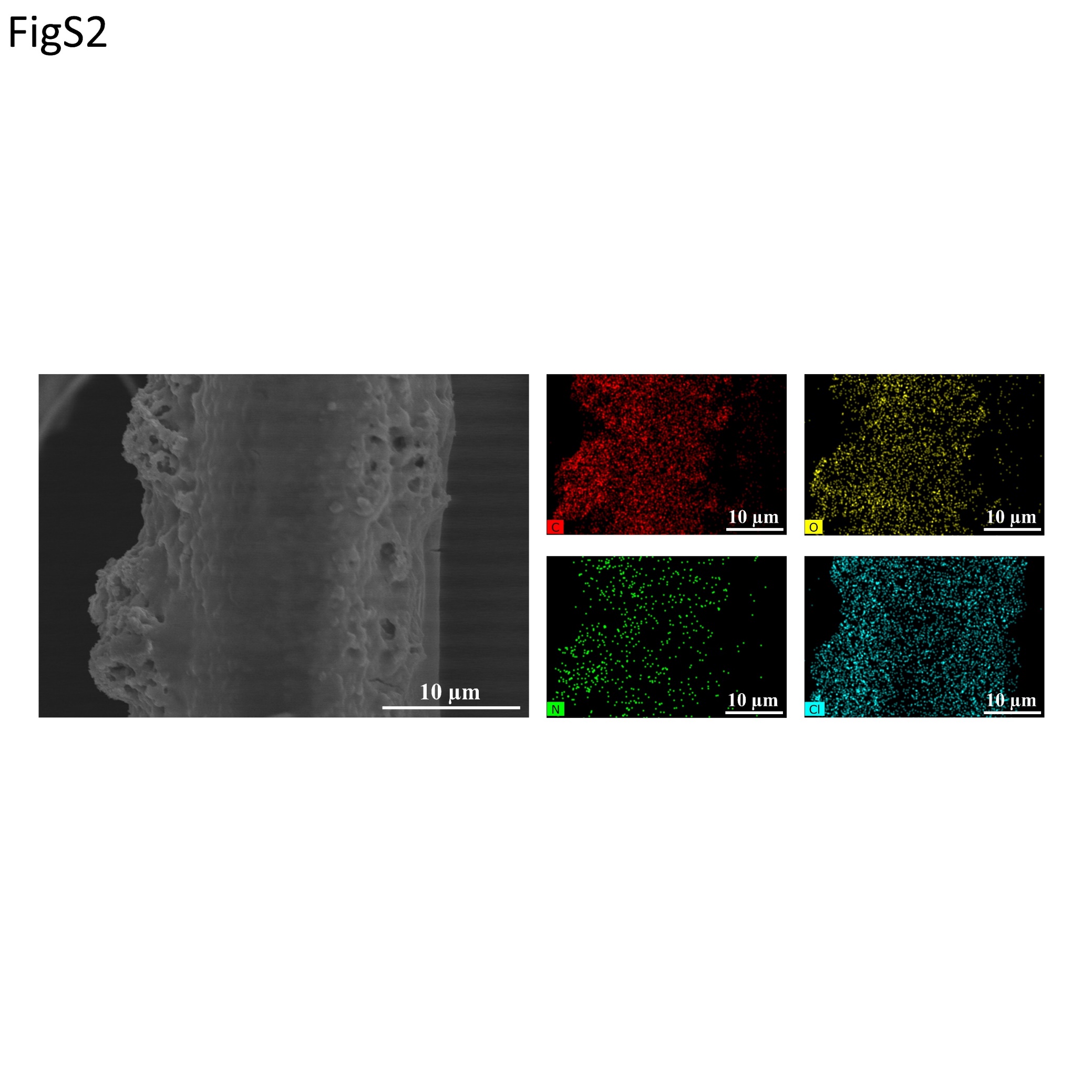


**Fig. S3** SEM mapping images of PPSF
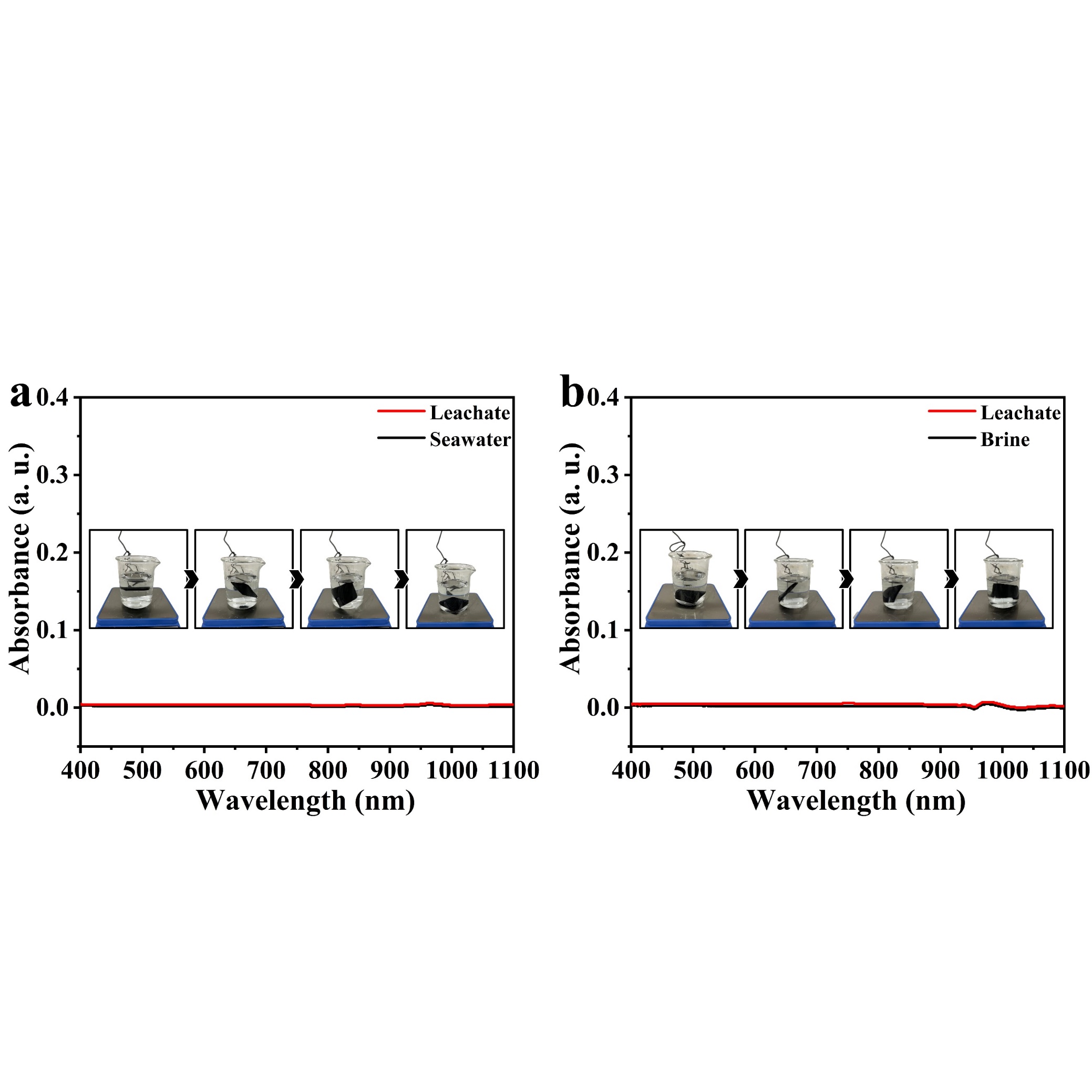


**Fig. S4** The photoabsorption of the leachate of the PPSF immersed in **a** seawater and **b** brine after 1 day. Inset: the immersed PPSF


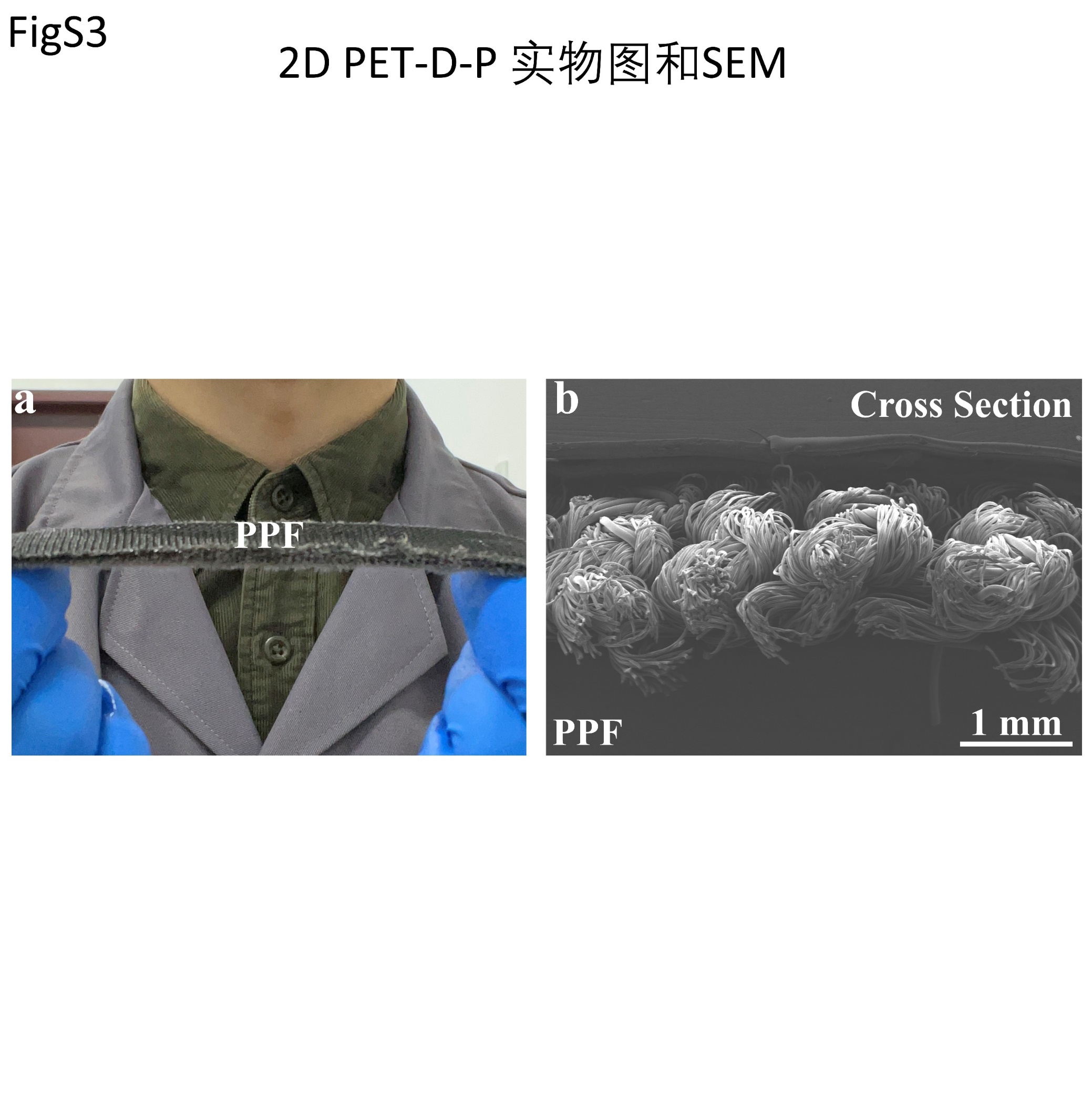


**Fig. S5 a** Digital photos and **b** cross section SEM images of the PPF


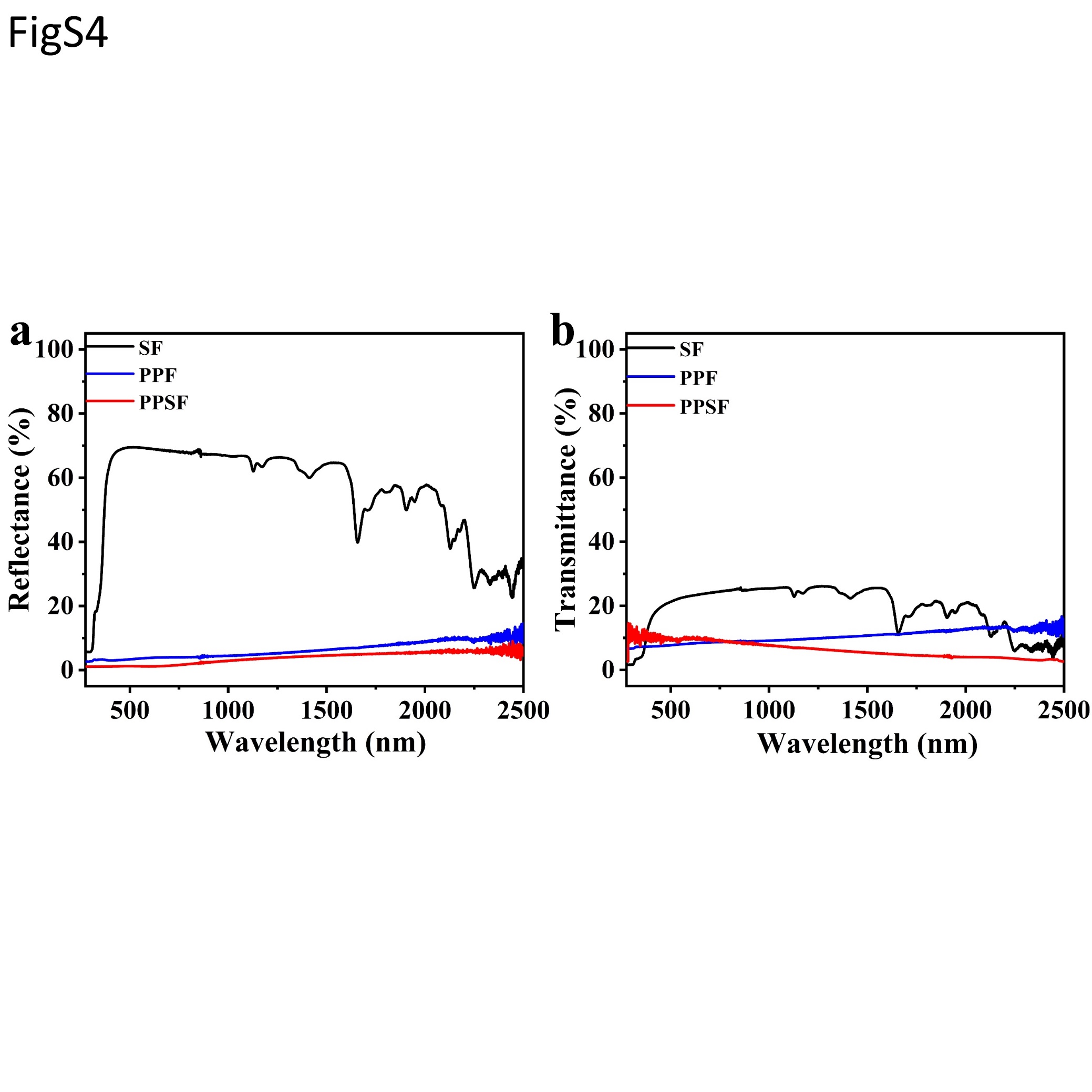


**Fig. S6 a** UV-Vis-IR reflectance spectra and **b** transmittance spectra of SF, PPF and PPSF


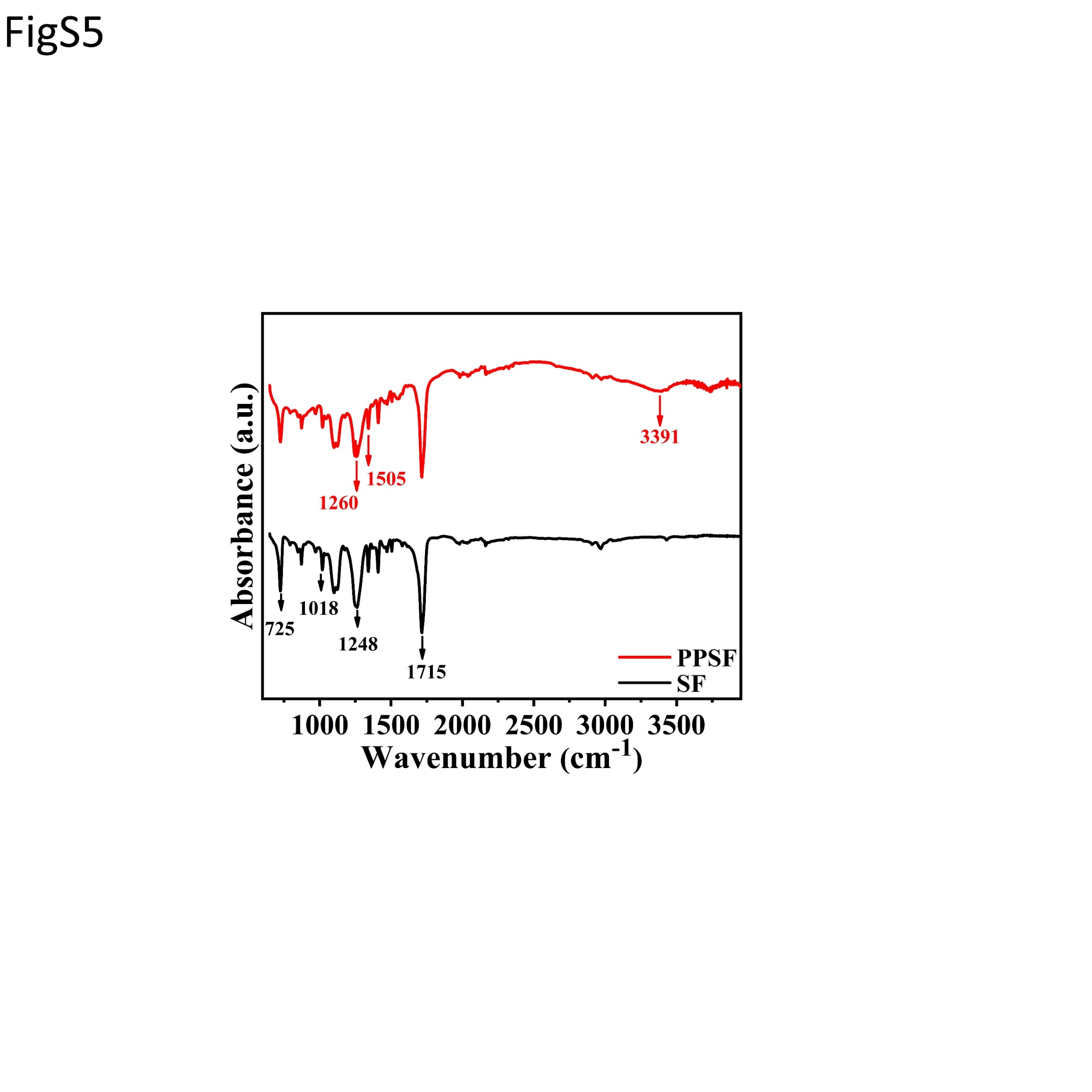


**Fig. S7** The FTIR spectra of SF and PPSF


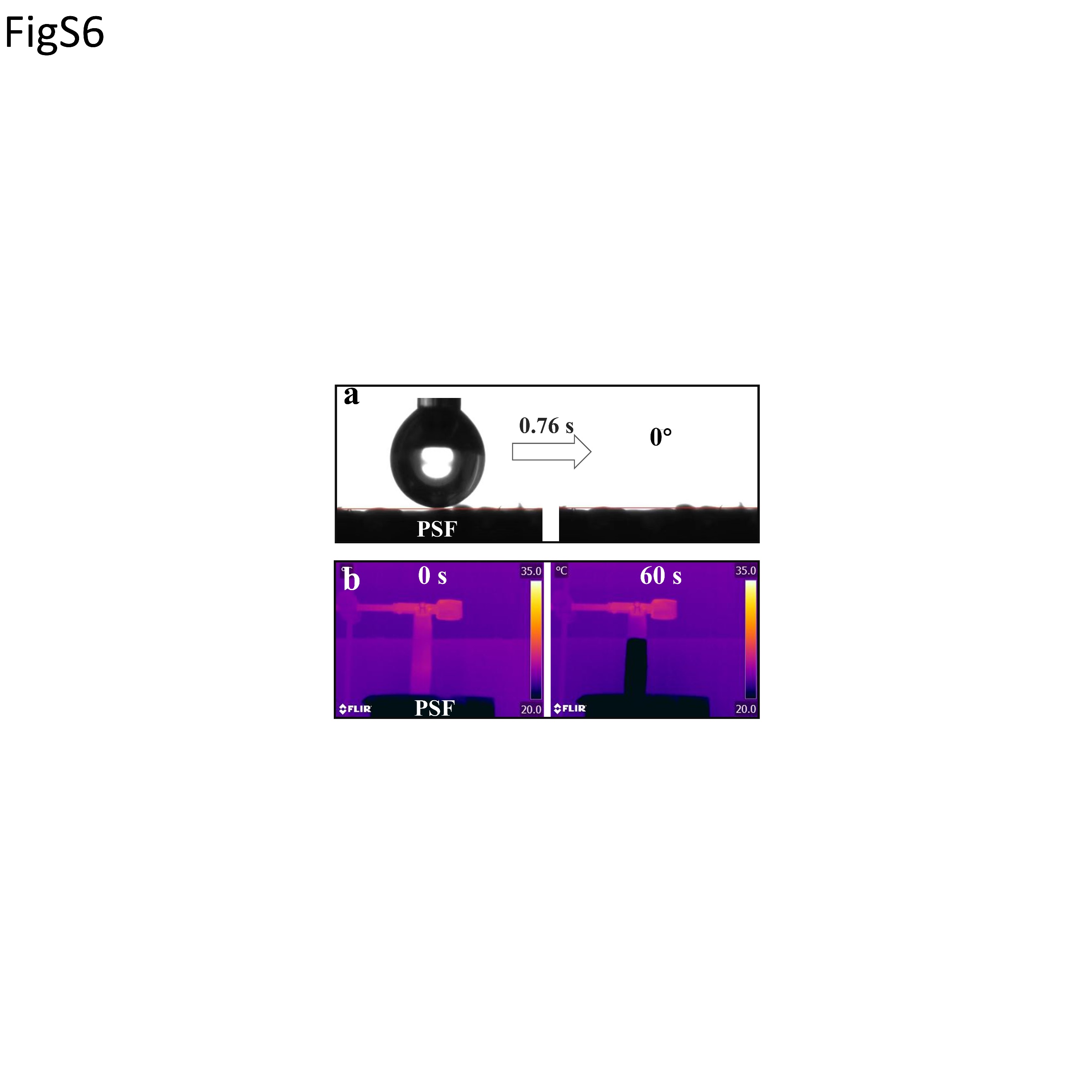


**Fig. S8 a** The shapes of a water droplet on the surface of PSF. **b** The water-soaking behavior of PSF


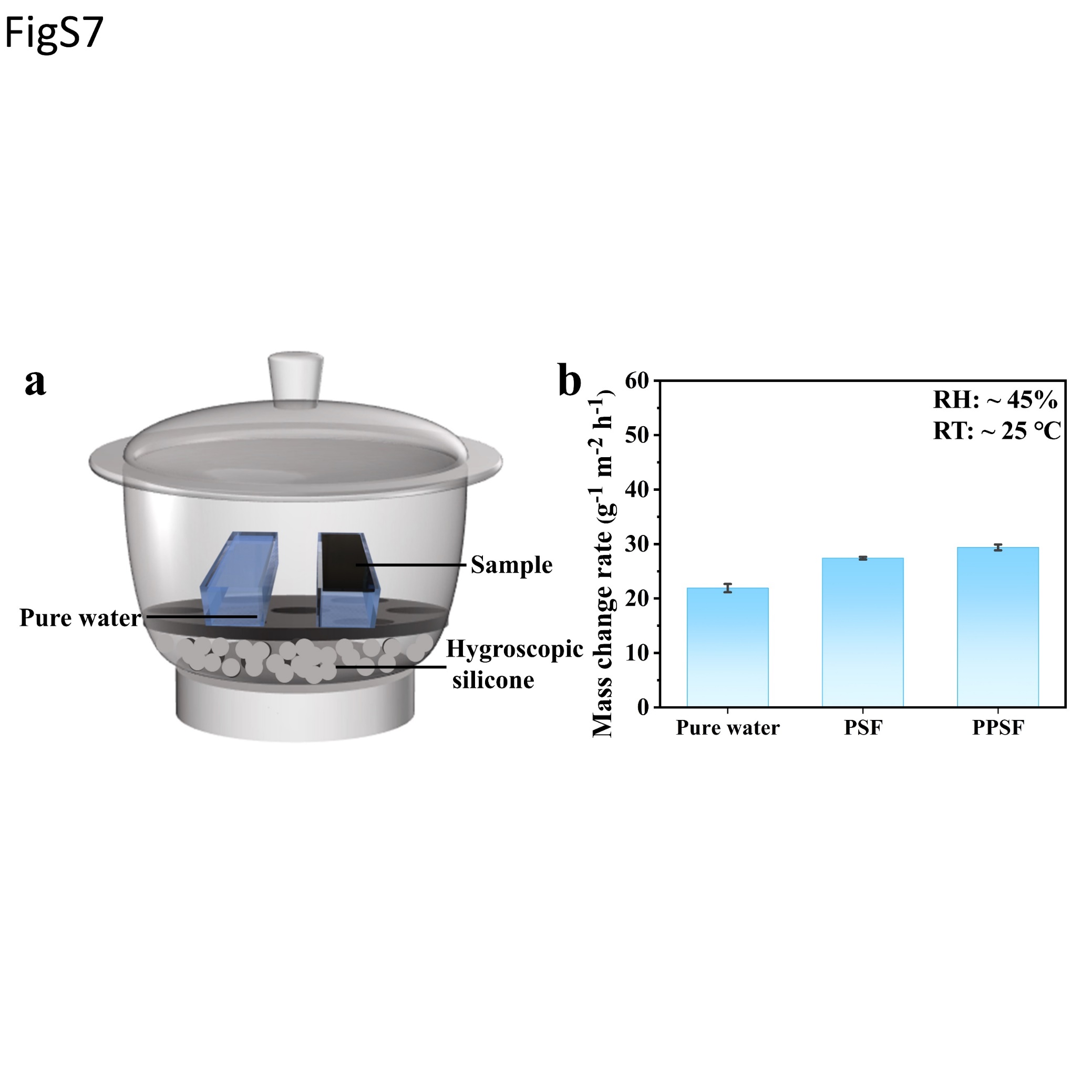


**Fig. S9 a** Schematic of the device with hygroscopic silicon at the bottom for testing the equivalent evaporation enthalpy. **b** Mass change rate in dark condition of bulk water and water in fabrics


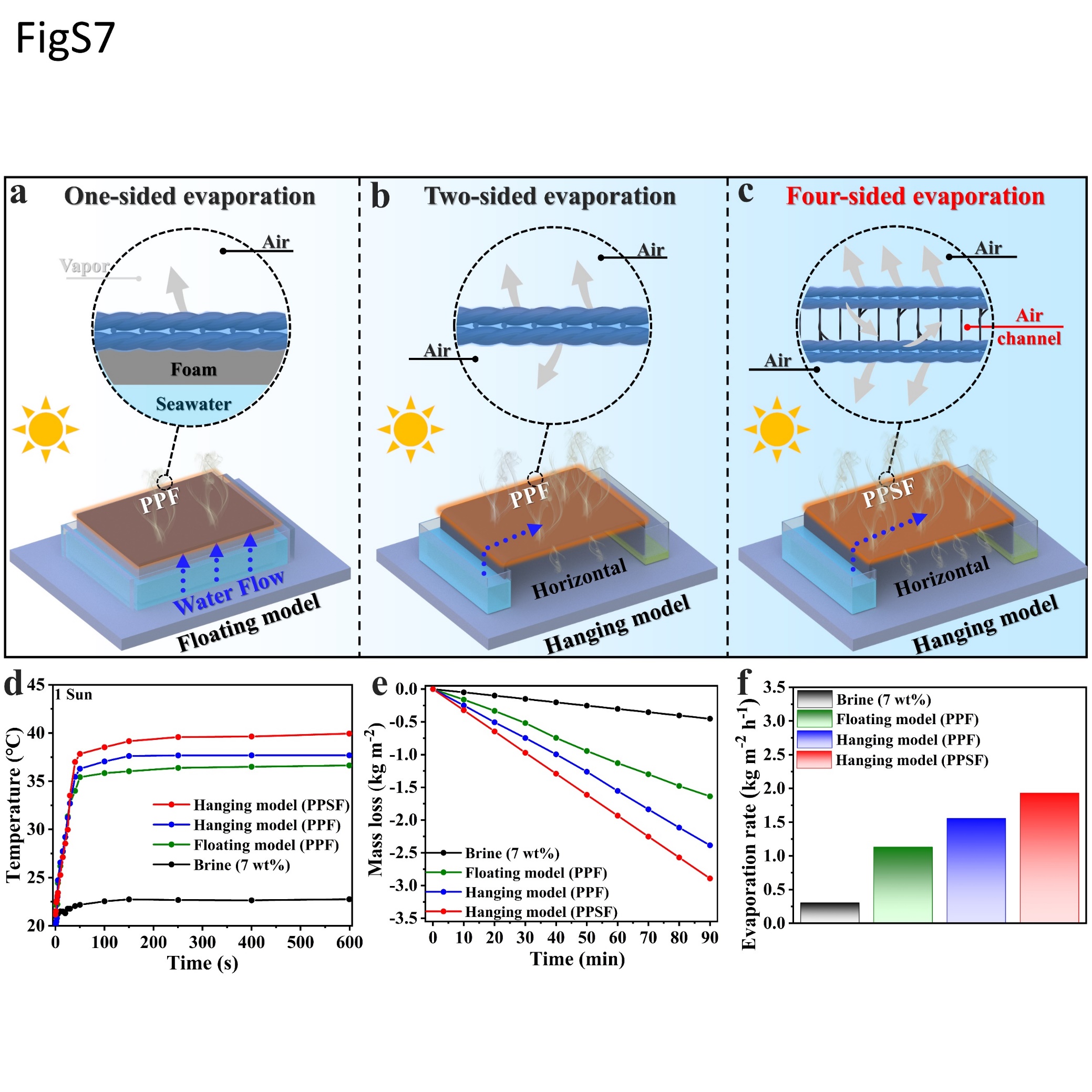


**Fig. S10 a** Traditional floating model with PPF for one-sided evaporation. **b** Horizontal hanging model with PPF fabric for two-sided evaporation. **c** Horizontal hanging model with PPSF fabric enabling four-sided evaporation. **d** Surface temperature curves, **e** evaporation mass, **f** evaporation rate of evaporation models under solar illumination (1 kW m^-2^)


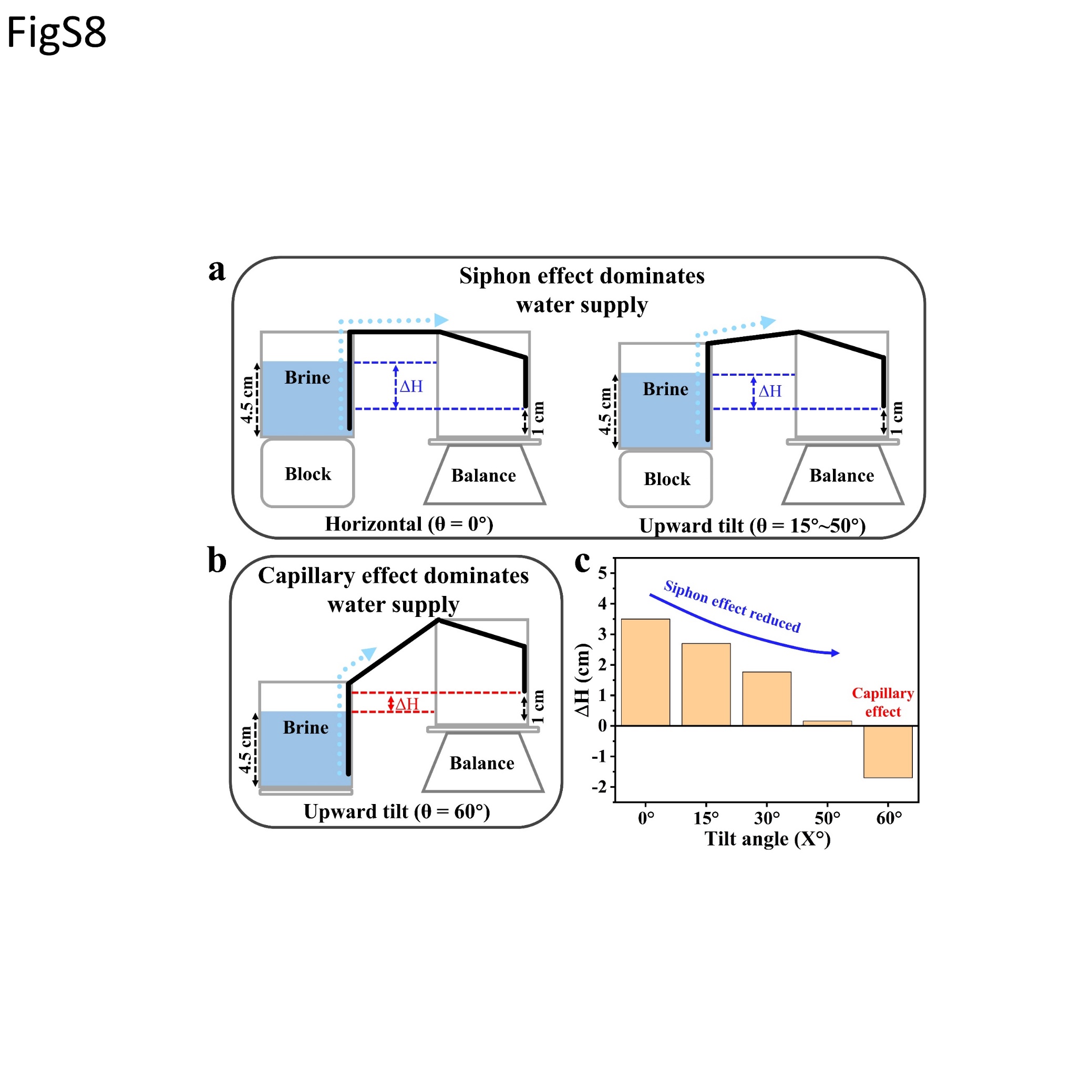


**Fig. S11 a** Schematic of siphon effect dominates water supply, when the tilt angle at from 0° to 50°. **b** Schematic of capillary effect dominates water supply, when the tilt angle at 60°. **c**

Effective head (ΔH, the height difference between the brine level in the water supply tank and the fabric end in the collection tank) of hanging model at different tilt angle.


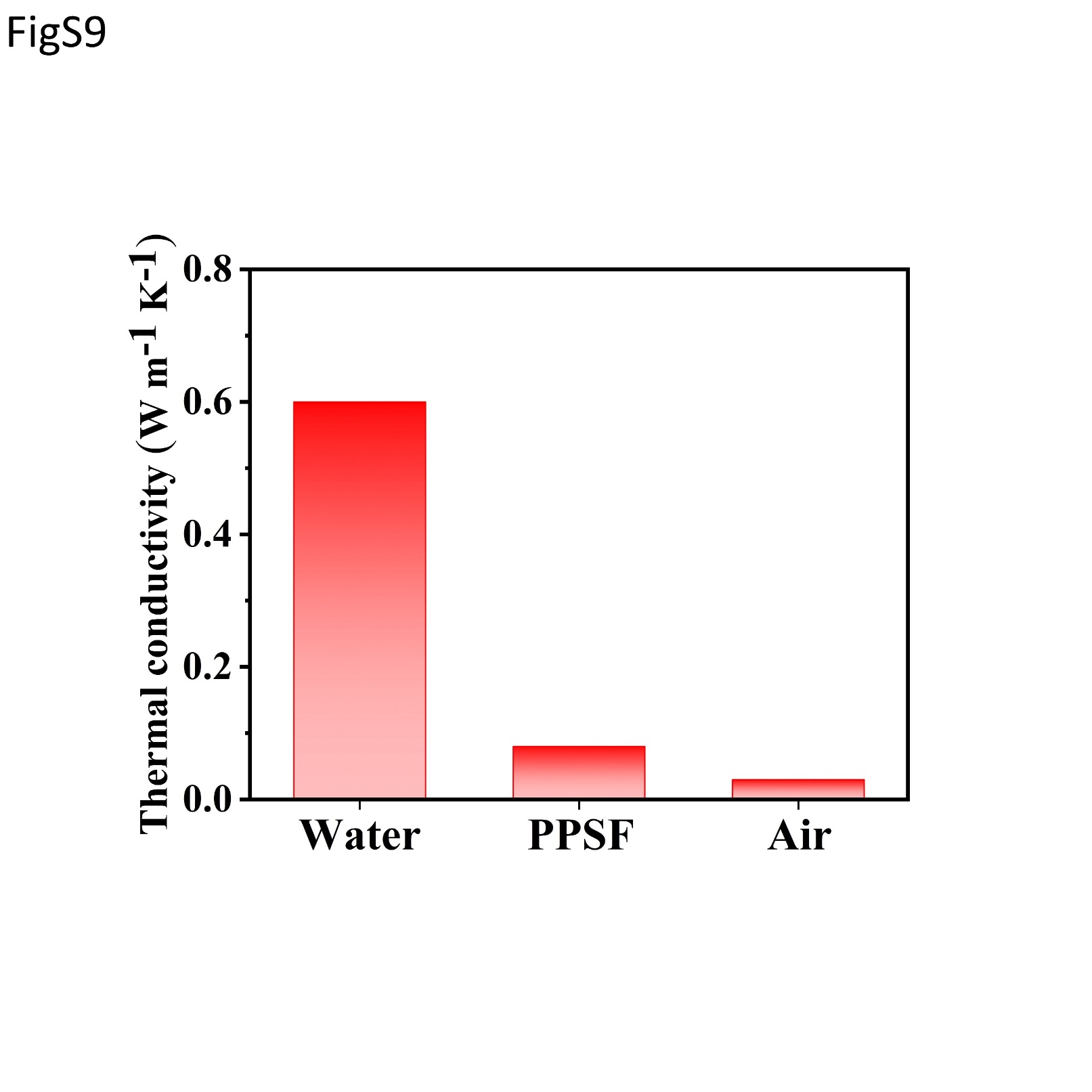


**Fig. S12** Thermal conductivity of Water, PPSF (in the dry state) and air


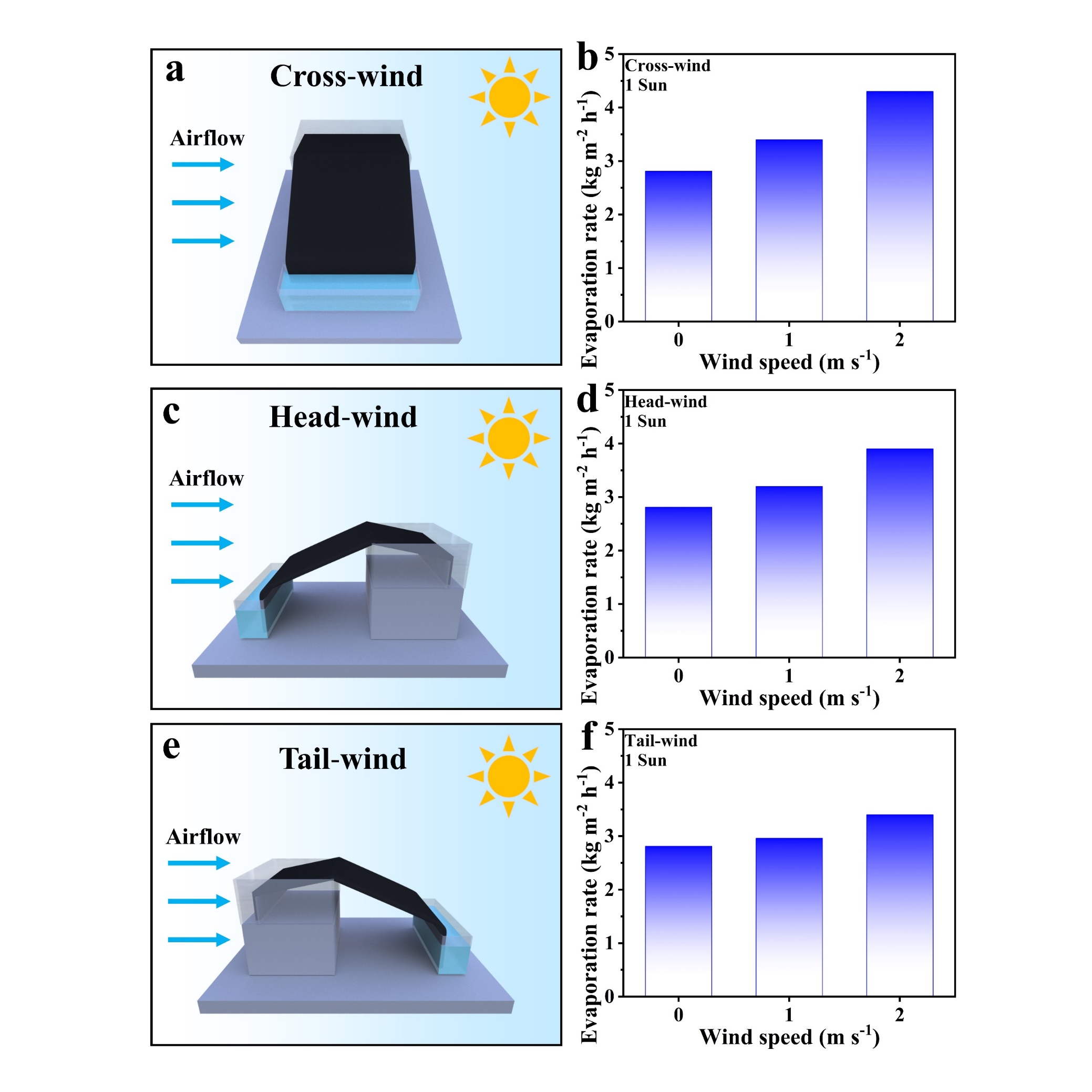


**Fig. S13** Influence of airflow orientation and velocity (0, 1, 2 m s⁻¹) on the upward hanging evaporator under solar illumination (1 kW m^-2^): **a, b** Cross-wind, **c, d** Head-wind, and **e, f** Tail-wind


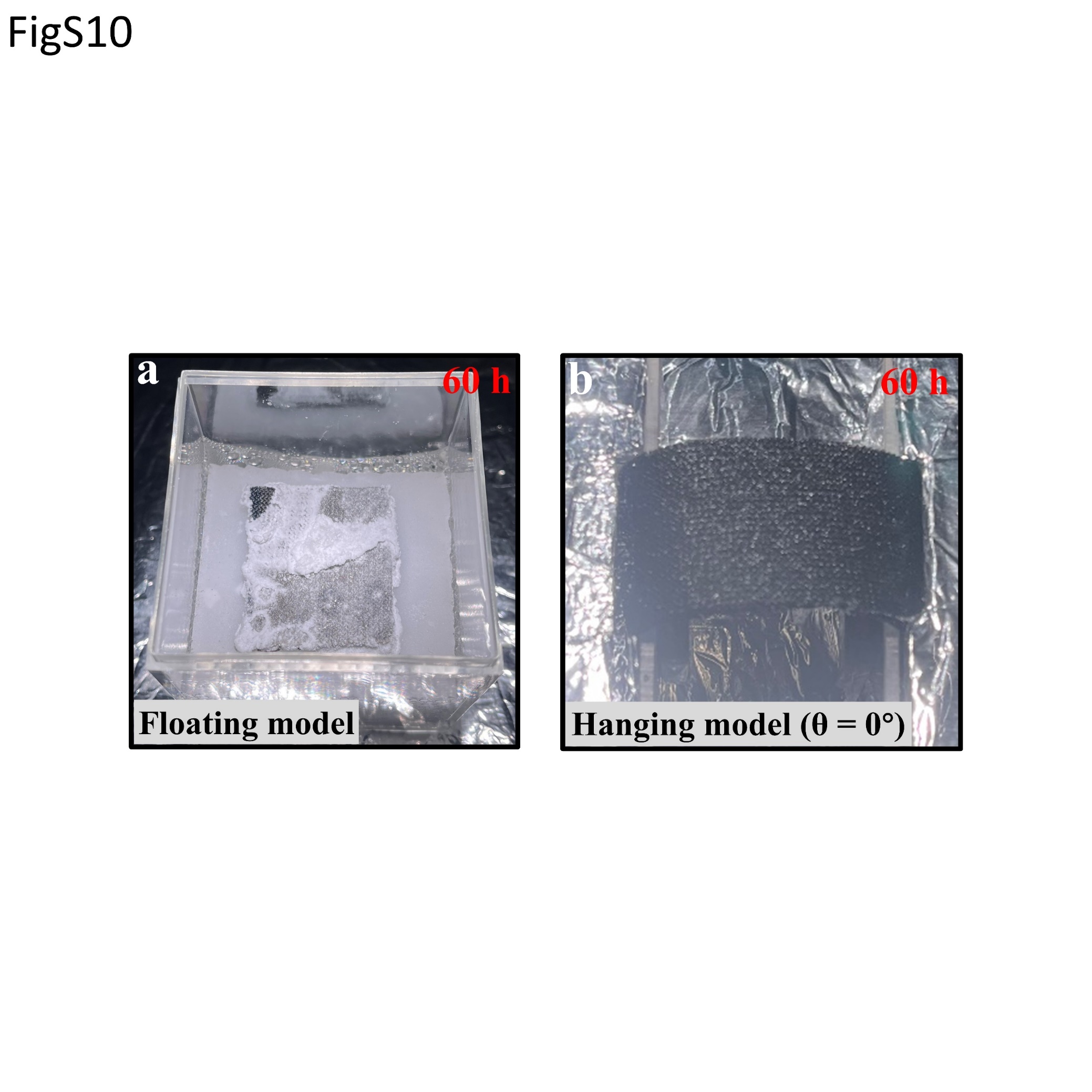


**Fig. S14** Photographs of the **a** floating and **b** horizontal hanging model (θ = 0°) during the long-term evaporation of brine solution (7 wt%) at the 60th hour


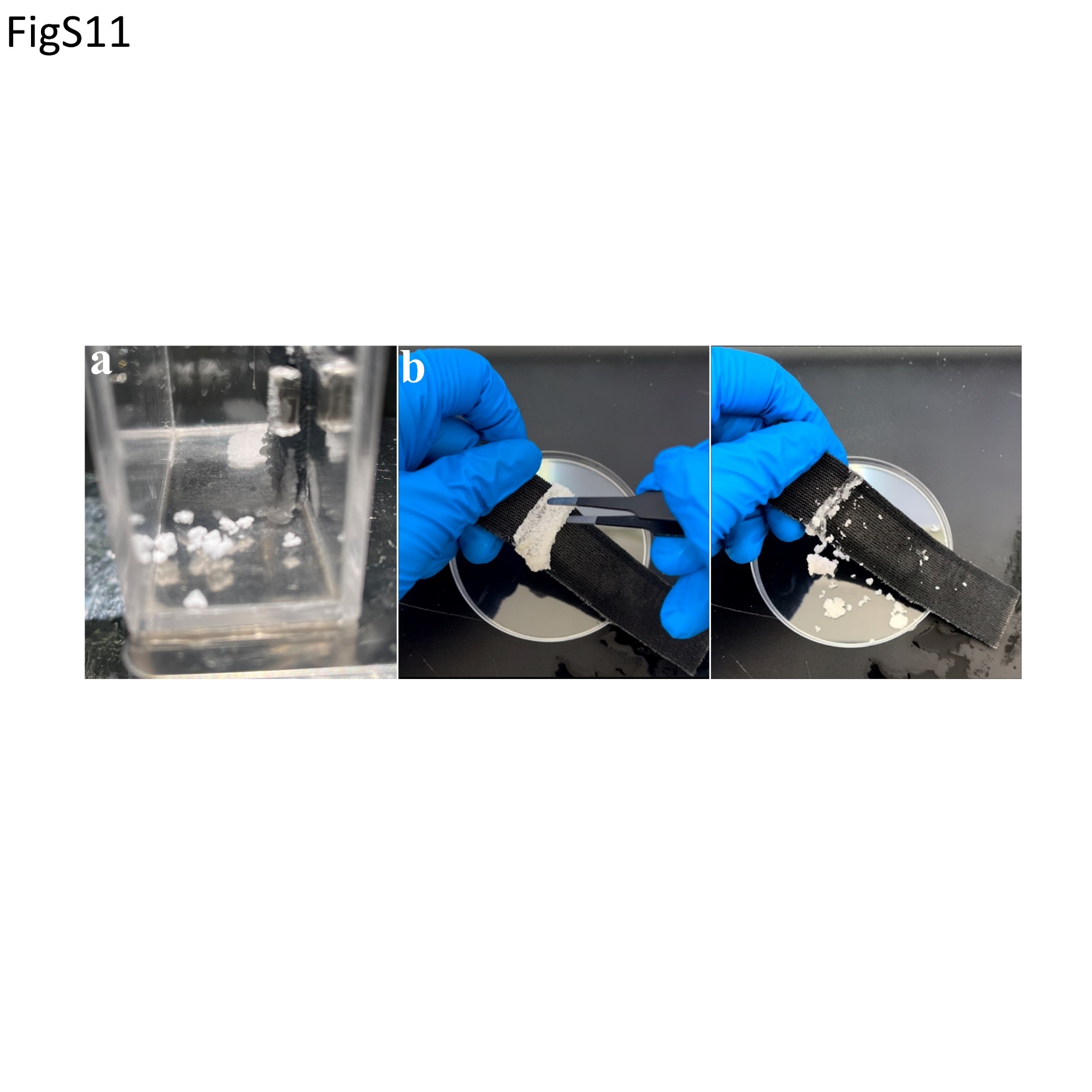


**Fig. S15** Photographs showing the salt collection by **a** natural drop-off or **b** manual scraping


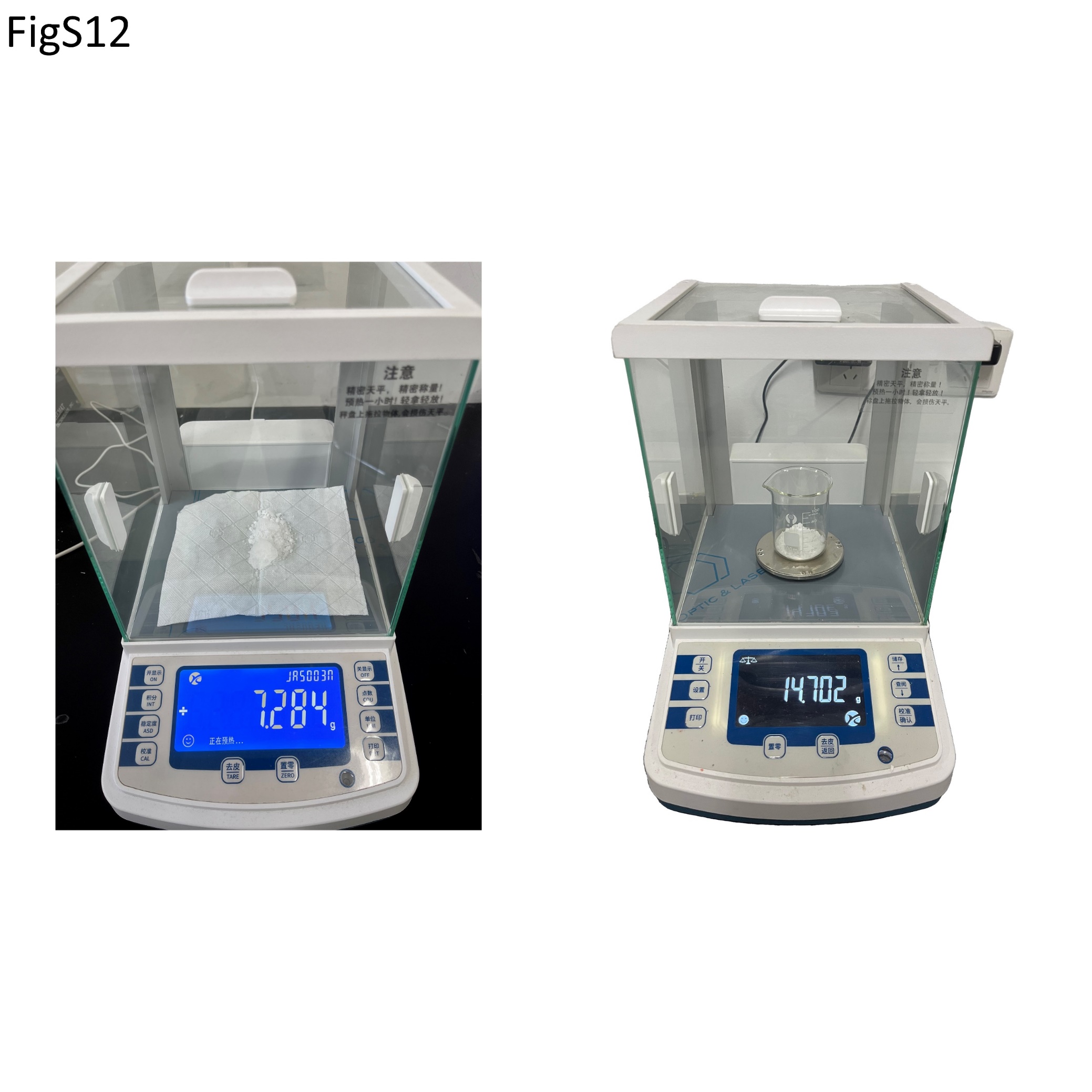


**Fig. S16** The upward hanging model evaporator collected 7.284 g of salt crystals


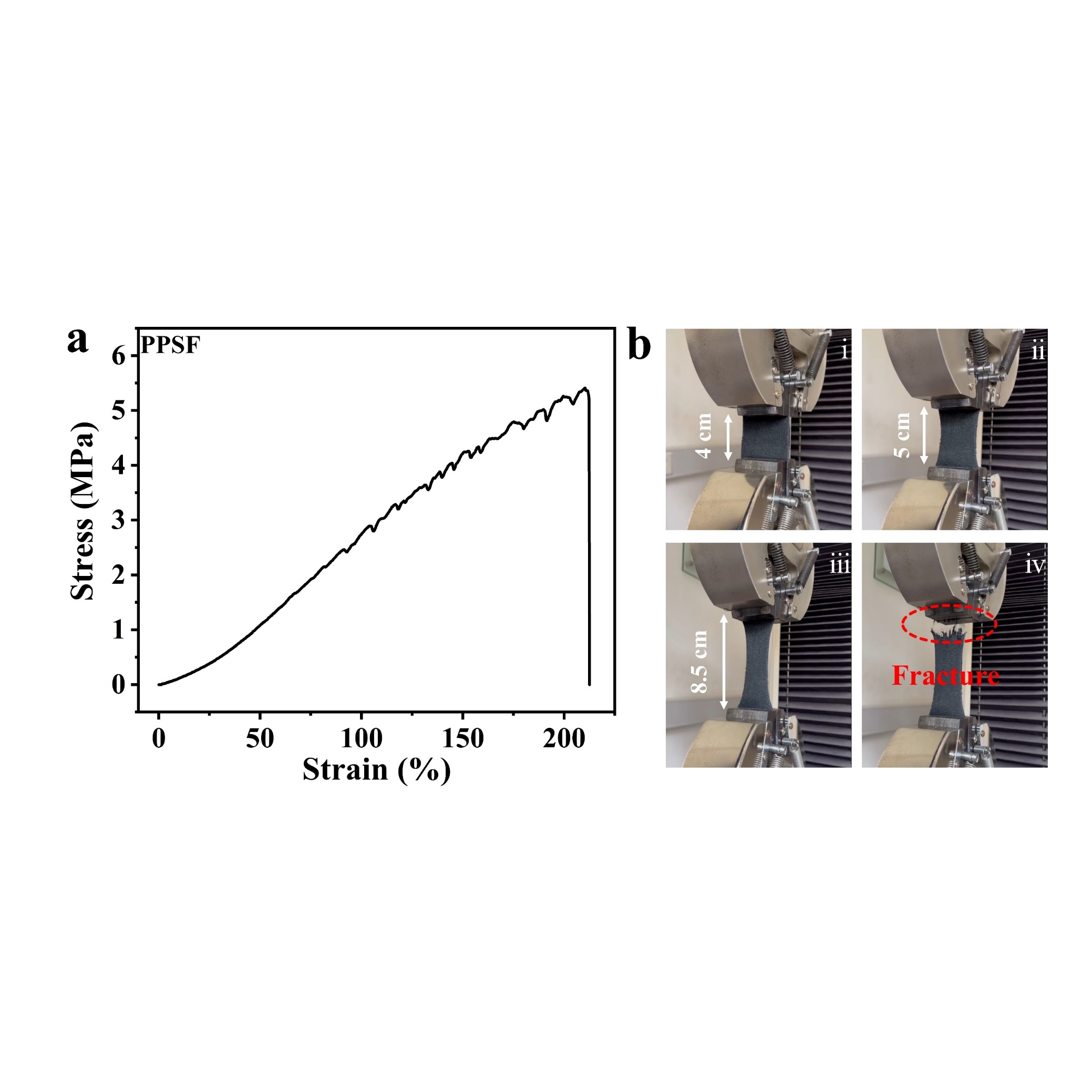


**Fig. S17** **a** Stress-strain curve and **b** photos corresponding the stretching process of PPSF after fourteen consecutive days of operation


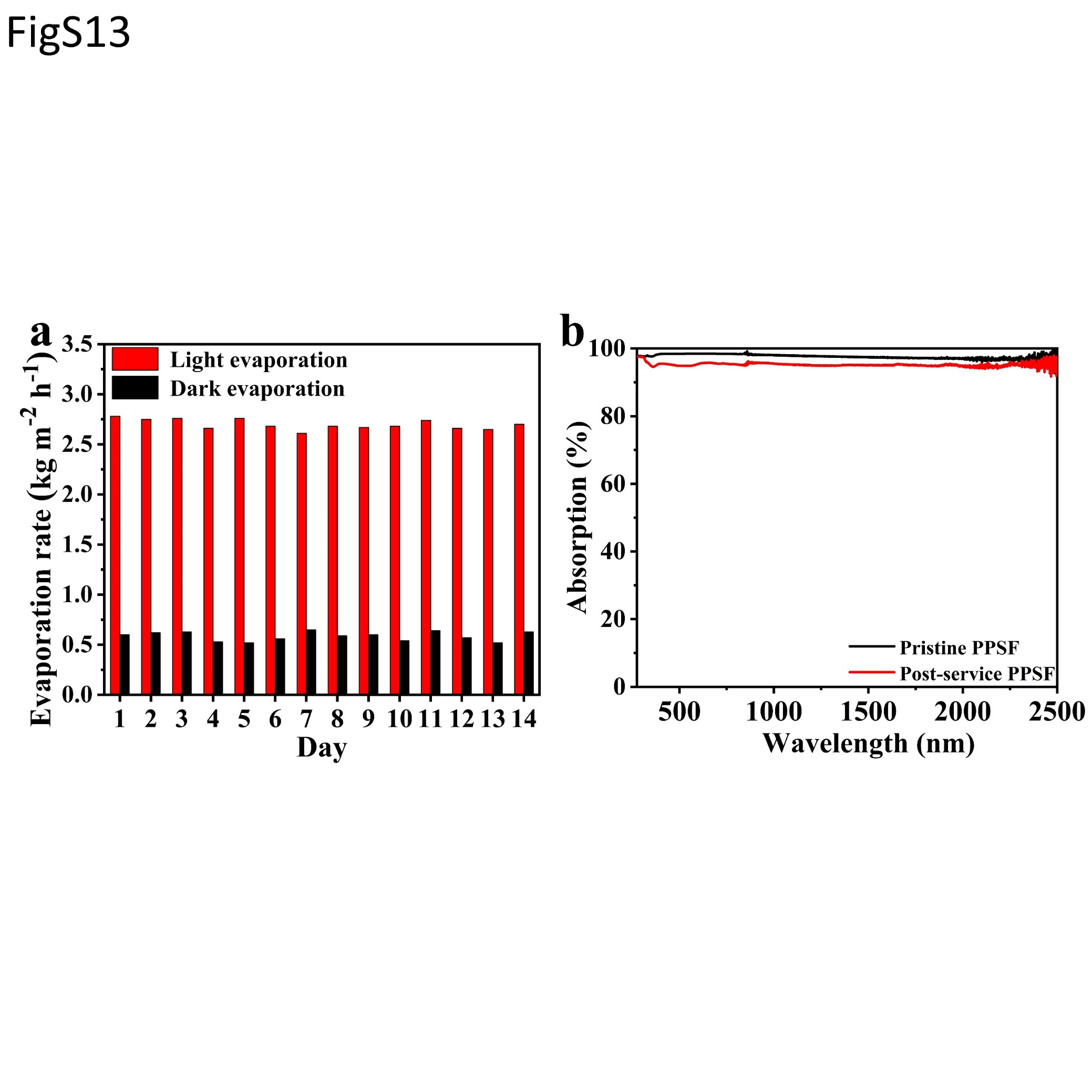


**Fig. S18 a** Long-term evaporation of upward hanging (θ = 52°) models during 14 consecutive days using brine solution (7 wt%), **b** UV–vis-IR spectra of pristine PPSF and post-service PPSF


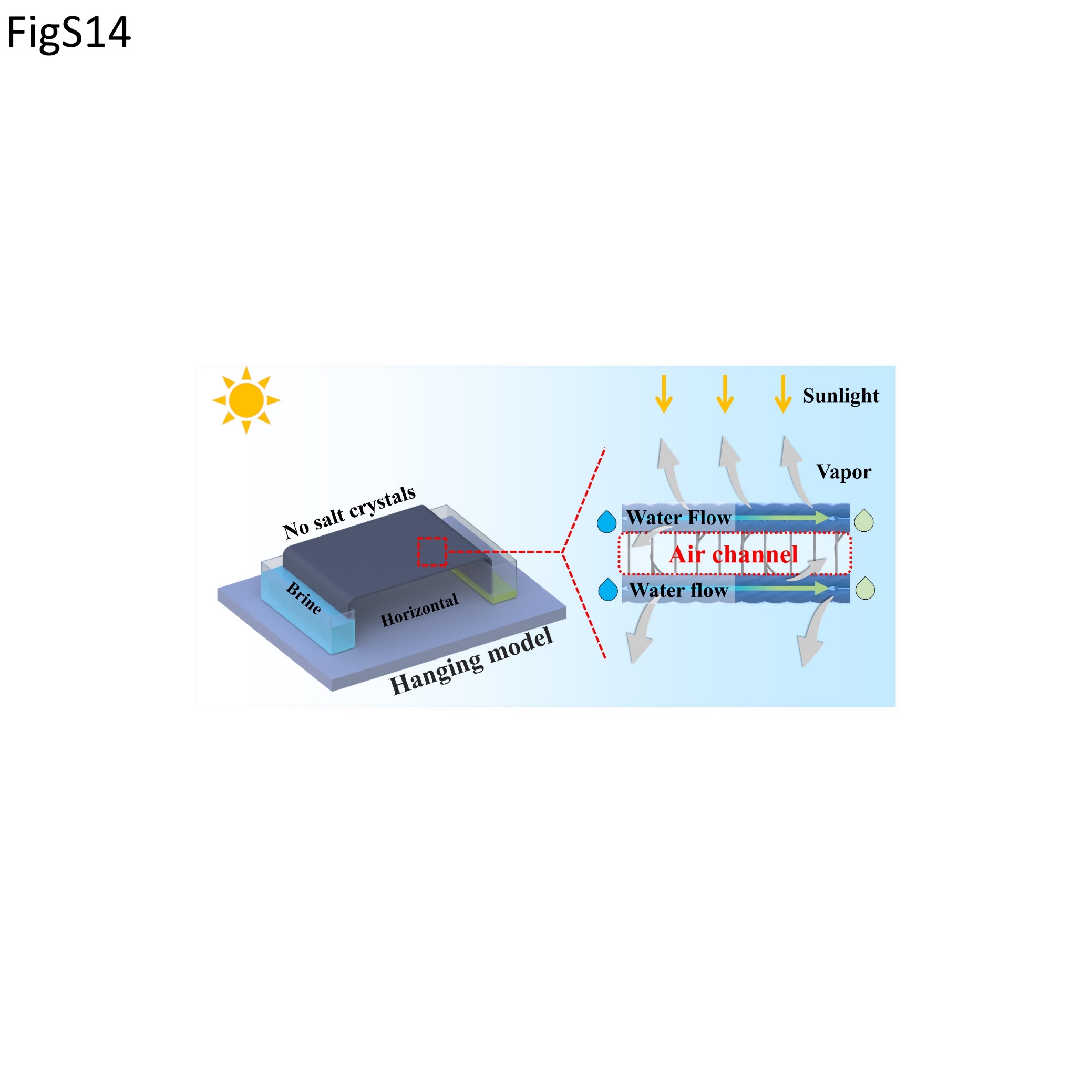


**Fig. S19** Schematic illustration of the hanging model (θ = 0°) achieving salt-free evaporation


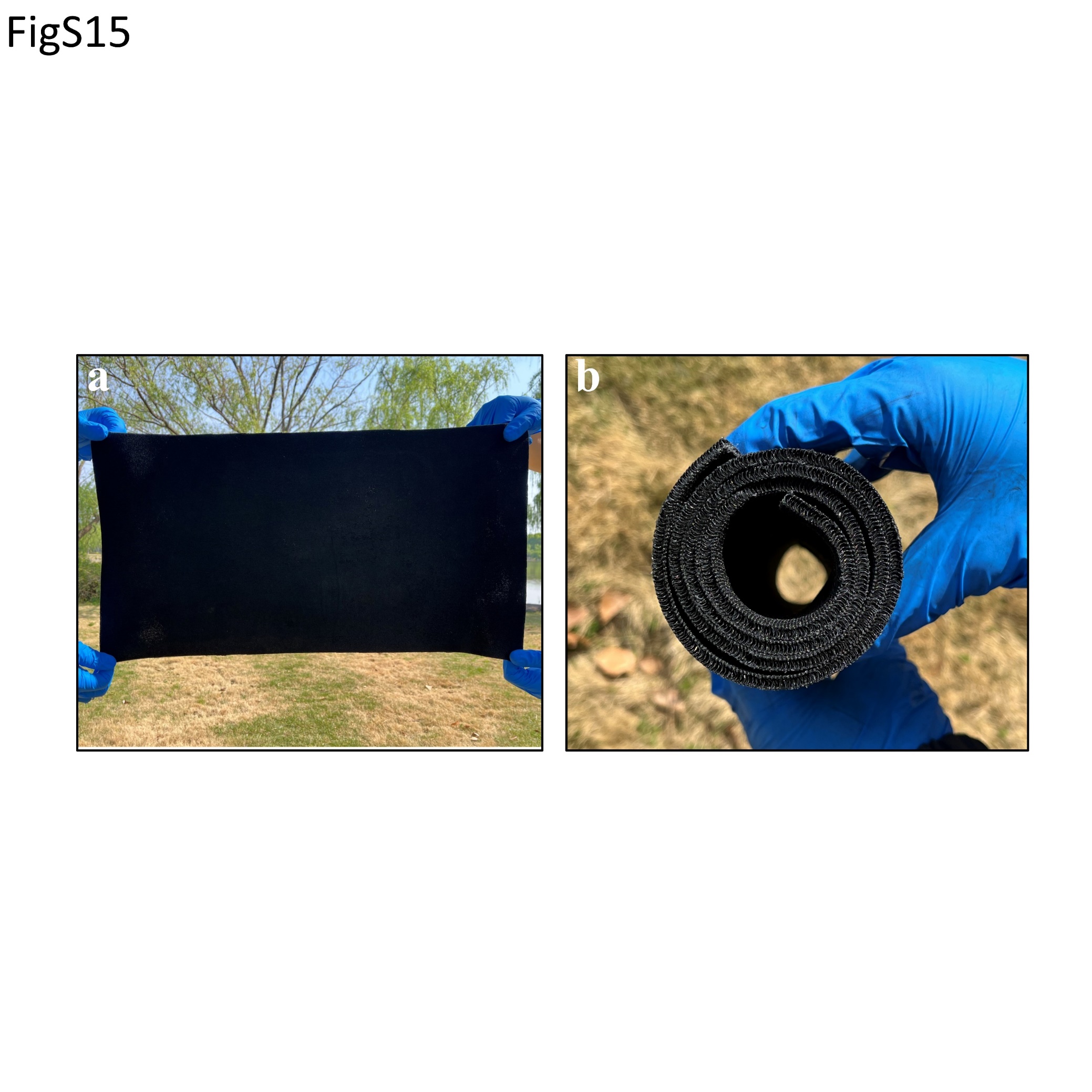


**Fig. S20 a** Photos and **b** flexible display of a large area (30 cm × 80 cm) of PPSF


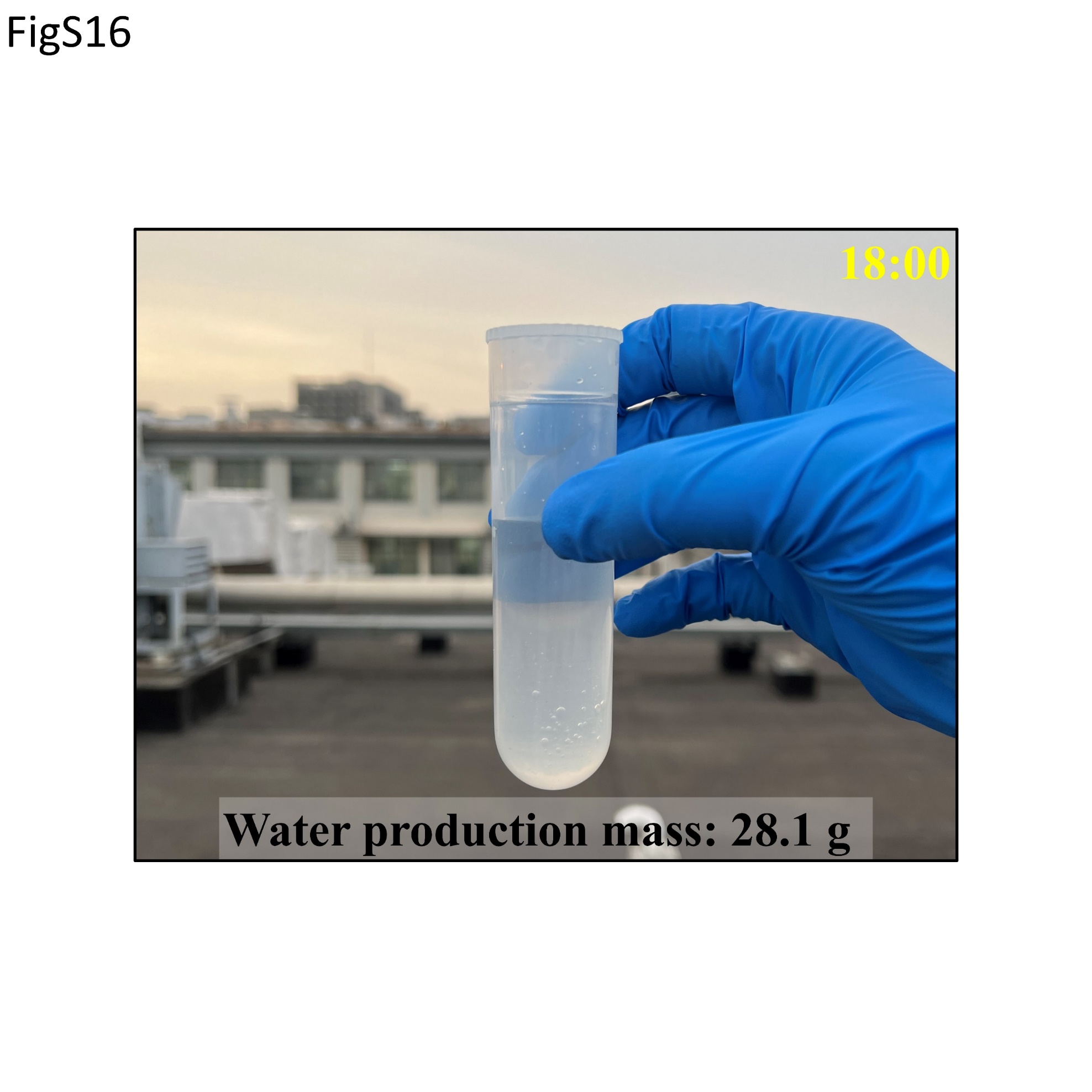


**Fig. S21** Photograph of the freshwater collected from 8:00 to 18:00 on July 3, 2024

**Supplementary References**

1. C.O. Popiel, J. Wojtkowiak, Simple formulas for thermophysical properties of liquid water for heat transfer calculations (from 0 to 150°c) (vol 19, pg 87, 1998). Heat Transf. Eng. **20**, 97-97 (1999). <https://doi.org/10.1080/01457639808939929>
2. X. Wu, Z. Q. Wu, Y. D. Wang, T. Gao, Q. Li et al., All-cold evaporation under one sun with zero energy loss by using a heatsink inspired solar evaporator. Adv. Sci. **8**, 202002501 (2021). <https://doi.org/10.1002/advs.202002501>
3. M.A. Abdelsalam, M. Sajjad, A. Raza, F. Almarzooqi, T.J. Zhang, Sustainable biomimetic solar distillation with edge crystallization for passive salt collection and zero brine discharge. Nat. Commun. **15**, 874 (2024). <https://doi.org/10.1038/s41467-024-45108-2>
